# Protective Effects of Magnesium Glycyrrhizinate on Methotrexate-Induced Hepatotoxicity and Intestinal Toxicity May Be by Reducing COX-2

**DOI:** 10.3389/fphar.2019.00119

**Published:** 2019-03-25

**Authors:** Yuzhu Cao, Hang Shi, Zhiguang Sun, Jiawei Wu, Yawen Xia, Yufei Wang, Yuanyuan Wu, Xiaoman Li, Wenxing Chen, Aiyun Wang, Yin Lu

**Affiliations:** ^1^School of Pharmacy, Nanjing University of Chinese Medicine, Nanjing, China; ^2^Department of The First College, Nanjing University of Chinese Medicine, Nanjing, China; ^3^Jiangsu Key Laboratory for Pharmacology and Safety Evaluation of Chinese Materia Medica, Nanjing University of Chinese Medicine, Nanjing, China; ^4^Jiangsu Collaborative Innovation Center of Traditional Chinese Medicine (TCM) Prevention and Treatment of Tumor, Nanjing University of Chinese Medicine, Nanjing, China

**Keywords:** magnesium isoglycyrrhizinate, methotrexate, hepatotoxicity, intestinal damage, inflammation, COX-2, ZO-1, Cx43

## Abstract

Magnesium isoglycyrrhizinate (MgIG), which has been widely employed to treat chronic hepatitis, is synthesized from 18-β glycyrrhizic acid, a main component of traditional Chinese medicine Glycyrrhiza uralensis Fisch. Although the protective effects of MgIG on methotrexate (MTX)-induced liver toxicity have been well-documented, the underlying mechanism remains elusive. MTX was initially used to treat pediatric acute leukemia, and has been widely applied to psoriasis therapy. However, its clinical applications are limited due to hepatotoxicity and intestinal toxicity. Herein, prophylactic administration of MgIG (9 and 18 mg/kg/day) significantly reduced the levels of aspartate aminotransferase and alanine aminotransferase in the serum of rats receiving intravenous injection of MTX (20 mg/kg body weight). MgIG also attenuated MTX-induced hepatic fibrosis. Moreover, it better protected against MTX-induced hepatocyte apoptosis and decreased the serum level of malondialdehyde than reduced glutathione (80 mg/kg/day) did. Interestingly, MTX-induced cyclooxygenase-2 (COX-2) expression, intestinal permeability and inflammation were attenuated after MgIG administration. In addition, MgIG (9 and 18 mg/kg) reduced MTX-induced colocalization of zonula occludens-1 (ZO-1) and connexin 43 (Cx43) in intestinal villi. In conclusion, MgIG exerted beneficial effects on MTX-induced hepatotoxicity and intestinal damage, as a potentially eligible drug for alleviating the hepatic and intestinal side effects of MTX during chemotherapy.

## Introduction

Magnesium isoglycyrrhizinate (MgIG) is a novel α-isomer compound synthesized by isomerization and salification from 18 β-glycyrrhizic acid, a main component of traditional Chinese medicine Glycyrrhiza uralensis Fisch with well-known detoxifying effects (Gu et al., 2014). MgIG has mainly been used for treating chronic viral hepatitis, combating inflammation, protecting the liver cell membrane and improving liver functions (Xie et al., 2015). It is well-documented that MgIG was able to mitigate hepatotoxicity induced by lipopolysaccharide (Jiang et al., 2017b), ethanol (Lu et al., 2017), concanavalin A (Yang et al., 2016), cyclophosphamide (Jiang et al., 2017a), paclitaxel (Chen et al., 2014), and free fatty acid (Cheng et al., 2009).

Methotrexate (MTX) was first used to treat pediatric acute leukemia, and has been applied to psoriasis and rheumatoid arthritis therapies worldwide (Bastian et al., 2011; Rajitha et al., 2017; Jenko et al., 2018). However, it has a well-defined toxicity profile of which hepatotoxicity has been considered to be the most important (Visser and van der Heijde, 2009; Conway and Carey, 2017). MTX can increase hepatic transaminase level, change liver histology, and lead to fibrosis and cirrhosis (Conway and Carey, 2017). The clinical applications of MTX are limited owing to hepatotoxicity (Visser and van der Heijde, 2009).

Accumulating evidence has verified the protective effects of MgIG on MTX-induced liver toxicity (Wu et al., 2017; Kong, 2018). Nevertheless, the underlying mechanism is still largely unknown. MTX is a stable derivative of aminopterin, the first folate antimetabolite, which can inhibit the synthesis of purines and pyrimidine, and decrease DNA synthesis, repair and cellular replication (Philips et al., 1973). Additionally, MTX damages the intestinal mucosa, thereby disrupting the intestinal barrier functionality and allowing bacterial translocation to the liver to induce hepatotoxicity (Song et al., 2006; Duman et al., 2013). The increase in intestinal permeability is positively correlated with diarrhea, and MTX-induced high permeability significantly participates in liver inflammation (Tran-Minh et al., 2017). Interestingly, Hu et al. (2003) demonstrated that MgIG is mainly excreted into bile in unchanged form. Thereby motivated, we herein studied the effects and the underlying mechanisms of MgIG on methotrexate-induced hepatotoxicity and intestinal toxicity.

## Materials and Methods

### Animal Treatment and Experimental Design

Male Wistar (SCXK 2012-0001) rats weighing 180–220 g were obtained from Beijing Vital River Laboratory Animal Technology Co., Ltd (Beijing, China). All animal procedures were performed according to the guidelines for the Institutional Animal Ethics Committee and approved by the Institutional Animal Committee of Nanjing University of Chinese Medicine (Supplementary Data Sheets S5, S6). The rats were housed under standard laboratory conditions for approximately one week before experimentation. The rats were injected intravenously with a single dose of MTX (20 mg/kg) (Miyazono et al., 2004; Khalifa et al., 2017).

The recommended daily dosage of MgIG for clinical use is 100–200 mg/d, so we herein selected the best dosages of 9 and 18 mg/kg/d for rats. Thirty rats were randomly divided into five groups: (1) Control group, (2) MTX group (20 mg/kg, i.v., once), (3) MTX (20 mg/kg, i.v., once) + glutathione (GSH, 80 mg/kg/d, i.v., injection using vial, seven consecutive days) group, (4) MTX (20 mg/kg, i.v., once)+ MgIG (9 mg/kg/d, i.v., seven consecutive days) group, and (5) MTX (20 mg/kg, i.v., once) + MgIG (18 mg/kg/d, i.v., seven consecutive days) group. On the first day, the rats were administered with MTX. MgIG/GSH was given through the caudal vein every day, and the control group was treated with an equal volume of saline. After one week, the rats were anesthetized by urethane (1.0 g/kg) intraperitoneally, and blood was collected from the abdominal aorta, left still for 4 h at room temperature and centrifuged to separate the serum. The levels of aspartate aminotransferase (AST) and alanine aminotransferase (ALT)-related liver function parameters were measured.

### Chemicals and Reagents

MgIG (purity > 98%) was provided by Chia Tai Tianqing Pharmaceutical Group Co., Ltd. (China). GSH was purchased from Chongqing Pharmaceutical Co., Ltd. (China) and dissolved in 0.9% normal saline for *in vivo* rat treatments. MTX was purchased from Jiangsu Hengrui Medicine Co., Ltd. (China).

### Biochemical Measurement

Serum levels of AST and ALT were determined by Hitachi 7020 automatic blood biochemical analyzer (Tokyo, Japan). Malondialdehyde (MDA), superoxide dismutase (SOD), GSH and glutathione peroxidase (GPx) levels were measured using standard kits purchased from Nanjing Jiancheng Bioengineering Institute (China) by PerkinElmer EnSpire multifunctional microplate reader (Waltham Mass, United States).

### Western Blot Analysis

The protein extracts of liver tissue were separated by sodium dodecyl sulfate–polyacrylamide gel electrophoresis gels and then transferred to polyvinylidene difluoride membranes (Millipore, United States). Protein concentration was quantified by the BCA method using the BCA protein kit (Beyotime Institute of Biotechnology, China). The proteins were probed with cyclooxygenase-2 (COX-2), caspase-3, Bax, Bcl-2, PARP and cleaved-PARP (Cell Signaling Technology, United States; 1:1,000), and detected by enhanced chemiluminescence. Secondary antibodies were used at a dilution of 1:10000. Protein bands were imaged using Bio-Rad Bio-Spectrum Gel Imaging System (Hercules, CA, United States), and the intensities were quantified by Photoshop CS6 software (Adobe, United States). All protein bands were normalized to that of GAPDH protein.

### HE, Sirius Red and Immunohistochemical Staining

Liver and intestinal tissues were cut into small pieces and fixed in 10% neutral-buffered formalin for 24 h. Afterward, the tissue specimens were embedded in paraffin according to standard histological procedures. Tissue sections (thickness: 3 μm) were stained with HE. The liver damage severity was evaluated based on congestion of red blood cells (RBCs), inflammatory cell infiltration and pyknosis (Mehrzadi et al., 2018), and the mucosal damage severity was assessed as described previously to evaluate the parenchymal and stromal structures of liver tissues (Xian et al., 1999). The sections were observed under a light microscope, and the images were analyzed using ImageJ software (National Institutes of Health, Bethesda, MD, United States) The percentage (%) of intensity in sirius red staining was calculated with the following formula: fibrosis area/whole area × 100 The immunohistochemical assays of COX-2 (Cell Signaling Technology, United States; 1:500) and collagen I (Abcam, United States; 1:500) have been described before. The mean DAB intensity was quantified by Mantra Quantitative Pathology Workstation (PerkinElmer, United States).

### Terminal dUTP Nick End Labeling (TUNEL) Assay

DNA strand breaks were assessed by using one-step TUNEL apoptosis assay kit (Beyotime Institute of Biotechnology, China) following the manufacturer’s protocol. Nuclei were counterstained with 10 g/mL 4,6-diamidino-2-phenylindole (DAPI) (Beyotime Institute of Biotechnology, China). Cells stained by TUNEL were imaged and evaluated under Mantra fluorescent microscope (PerkinElmer, United States) at the excitation wavelength of 488 nm and the emission wavelength of 530 nm. Positive and total hepatocytes in each section were counted in 6 randomly selected high-power fields (× 200) under the light microscope, and the positive rates of TUNEL were calculated.

### Measurement of Intestinal Permeability

The rats were moved to metabolic cages to collect their 6 h urine after intragastric administration of a complex solution containing 120 mg lactulose and l80 mg/L mannitol on the seventh day after MgIG treatment. The urine was quantitatively analyzed by evaporative light-scattering detection (ELSD). Then 100 mg lactulose (Sigma Aldrich, United States) and 50 mg mannitol (Sigma-Aldrich, United States) were mixed in 2 mL of normal saline. The resulting test solution was administered orally. All urine samples were collected for 6 h and mixed thoroughly. A 2 mL sample was taken from the pooled urine and frozen at -20°C until analysis. Urinary lactulose and mannitol concentrations were measured by high-performance liquid chromatography-ELSD. NH_2_ column (No. C9815105191, Hanbon Science & Technology Co., Ltd., Huaian, China) was used with the mobile phase of 80% acetonitrile-water for gradient elution.

### Immunofluorescence Microscopy

Tissue sections (thickness: 3 μm) were subjected to immunofluorescence staining as previously reported. Briefly, paraffin sections of liver tissue (thickness: 3 mm) were deparaffinized and antigen-retrieved. Subsequently, the sections were blocked with 1% bovine serum albumin and incubated with antibodies at 4°C overnight. The unbound antibody was washed with PBS and then incubated with fluorescently labeled anti-rabbit or anti-mouse IgG antibody for 2 h at room temperature, followed by nuclear labeling with Hoechst, washing and fluorescence observation under Nikon fluorescence microscope (Tokyo, Japan).

### Statistical Analyses

Analyses were performed with GraphPad software (GraphPad Software Inc., La Jolla, CA, United States). Comparisons among multiple groups were conducted by one-way analysis of variance, and those between two groups were carried out with the Dunnett’s test. *P* < 0.05 was considered statistically significant.

## Results

### MgIG Reversed MTX-Induced Hepatotoxicity

**FIGURE 1:**
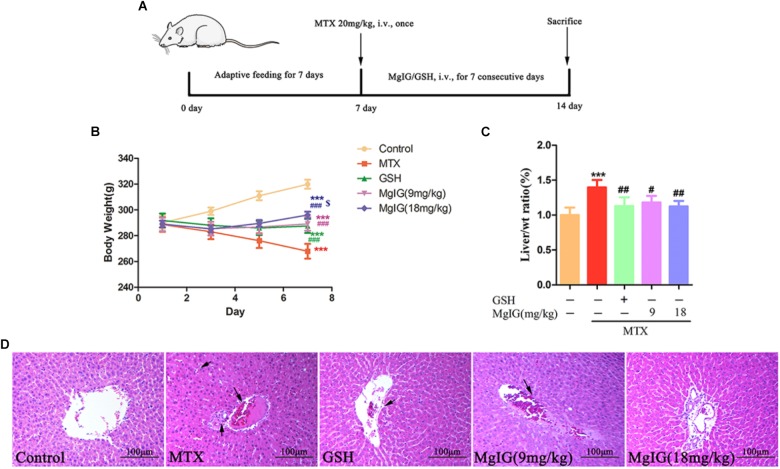
Effect of MgIG and MTX on body weight and liver weight/body weight ratio. **(A)** Experimental protocols for MTX-induced hepatotoxicity in rats pretreated with MgIG and GSH; **(B)** body weight; **(C)** liver weight/body weight ratio [Liver/wt ratio (%)]; **(D)** effects of treatment with MgIG and GSH on histopathological lesions in liver tissues following MTX-induced hepatotoxicity. Microscopic views from the pericentral zone of rat liver stained with HE are shown; magnifications: ×400. Values of body weight and liver weight are shown as mean ±*SD, n* = 6. Significance: Compared with control: ^∗^*P* < 0.05, ^∗∗^*P* < 0.01, ^∗∗∗^*P* < 0.001; compared with MTX group: ^#^*P* < 0.05, ^##^*P* < 0.01, ^###^*P* < 0.001; compared with GSH group: $*P* < 0.05. The arrow means congestion of RBCs, infiltration of inflammatory cells and pyknosis in liver.

**FIGURE 2:**
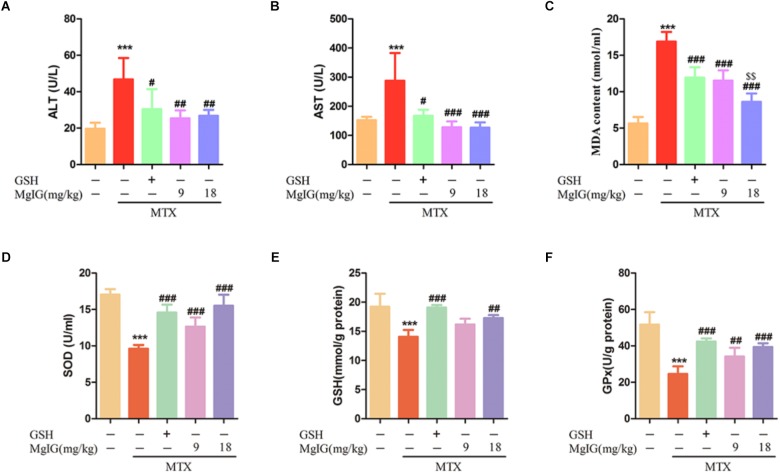
Effects of MgIG and MTX administration on serum and hepatic enzymatic levels **(A–D)** Effects of MgIG and MTX administration on serum ALT, AST, MDA and GSH levels; **(E,F)** effects of MgIG and MTX administration on activities of SOD and GPx. The results are shown as mean ±*SD* of six rats per group. Compared with control group: ^∗^*P* < 0.05, ^∗∗^*P* < 0.01, ^∗∗∗^*P* < 0.001; Compared with MTX group: ^#^*P* < 0.05, ^##^*P* < 0.01, ^###^*P* < 0.001; compared with GSH group: ^$$^*P* < 0.01.

Treatment with 9 and 18 mg/kg/d MgIG or 80 mg/kg/d GSH increased the body weight compared to that of the MTX group, and 18 mg/kg/d MgIG also significantly elevated the body weight compared to that of the 80 mg/kg/d GSH group (*P* < 0.05) (Figure 1A,B and Supplementary Tables S1, S2). The liver weight/body weight ratio of the MTX group significantly exceeded that of the MgIG or GSH group (Figure 1C). The MTX group had significantly higher levels of AST and ALT than those of the control group (Figure 2A,B). The rats pretreated with 9 and 18 mg/kg/d MgIG or 80 mg/kg/d GSH for seven consecutive days had significantly lower serum levels of ALT and AST than those of the MTX group (##*P* < 0.01, ##*P* < 0.01, #*P* < 0.05; ###*P* < 0.001, ###*P* < 0.001 and #*P* < 0.05, respectively). Furthermore, HE staining revealed that liver tissue of the MTX group suffered from congestion of RBCs, infiltration of inflammatory cells and pyknosis. Compared with the MTX group, the histopathological lesions were effectively mitigated after pretreatment with MgIG or GSH (Figure 1D and Supplementary Data Sheet S1).

### Effect of MgIG Administration on Hepatic Oxidative Stress Markers in MTX-Treated Rats

Oxidative stress plays a critical role in MTX-induced hepatotoxicity (Kelleni et al., 2016). To evaluate the antioxidant activity in liver tissue, the liver GSH level and activities of SOD and GPx were measured. Administration of MTX significantly decreased GSH level and activities of SOD and GPx compared to those of the control group. In contrast, the values significantly (*P* < 0.001) increased in MgIG and GSH groups (Figure 2D–F). Besides, pretreatment with MgIG at 9 and 18 mg/kg for seven consecutive days significantly decreased MDA level compared to that of the MTX group (*P* < 0.001) (Figure 2C).

### MgIG Alleviated MTX-Induced Apoptosis and Collagen Deposition in Hepatic Tissue

**FIGURE 3:**
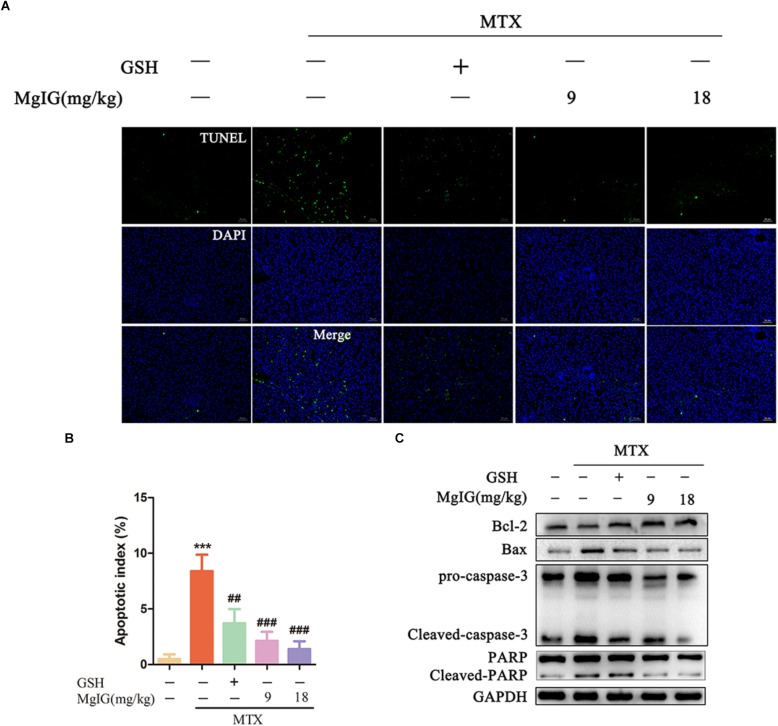
Effects of MgIG on MTX induced apoptosis in hepatic tissue: **(A)** Liver tissue sections from different treatment groups were stained with TUNEL (green) and counterstained with DAPI to localize the nuclei (blue), and colocalized green and blue cells in the same view indicated TUNEL-positive cells, scale bar = 50 μm; **(B)** semiquantitative analysis of TUNEL-positive cells, *n* = 6; **(C)** western blotting of liver caspase-3, cleaved-caspase-3, Bax, Bcl-2, PARP and cleaved-PARP protein expression. The results are shown as mean ±*SD, n* = 3. Compared with control group: ^∗^*P* < 0.05, ^∗∗^*P* < 0.01, ^∗∗∗^*P* < 0.001; compared with MTX group: ^#^*P* < 0.05, ^##^*P* < 0.01, ^###^*P* < 0.001.

Methotrexate can induce apoptosis and block the biosynthesis of cell DNA by inhibiting dihydrofolate reductase (Bajt et al., 2011; Xie et al., 2016). To investigate whether MgIG affected MTX-induced apoptosis in rat liver tissue, we first detected the apoptosis of hepatocytes by TUNEL assay and Western blot after MgIG and MTX exposures. The MTX group had significantly more TUNEL-positive cells than those in the control group (*P* < 0.001) (Figure 3A,B and Supplementary Table S3). The protein expression levels of caspase-3, cleaved caspase-3, Bax, Bcl-2, PARP and cleaved PARP were detected by Western blot. The expression levels of cleaved caspase-3, Bax and cleaved PARP proteins in the MTX group were up regulated compared with those of the control group, but the level of Bcl-2 decreased (Figure 3C and Supplementary Data Sheet S2). The percentage of TUNEL-positive cells in liver tissue significantly decreased after administration with 9 and 18 mg/kg MgIG (*P* < 0.05, *P* < 0.001, respectively). In addition, the protein levels of cleaved caspase-3, Bax and cleaved PARP in 9 and 18 mg/kg MgIG groups reduced compared with those of the MTX group, whereas the level of hepatic Bcl-2 increased.

**FIGURE 4:**
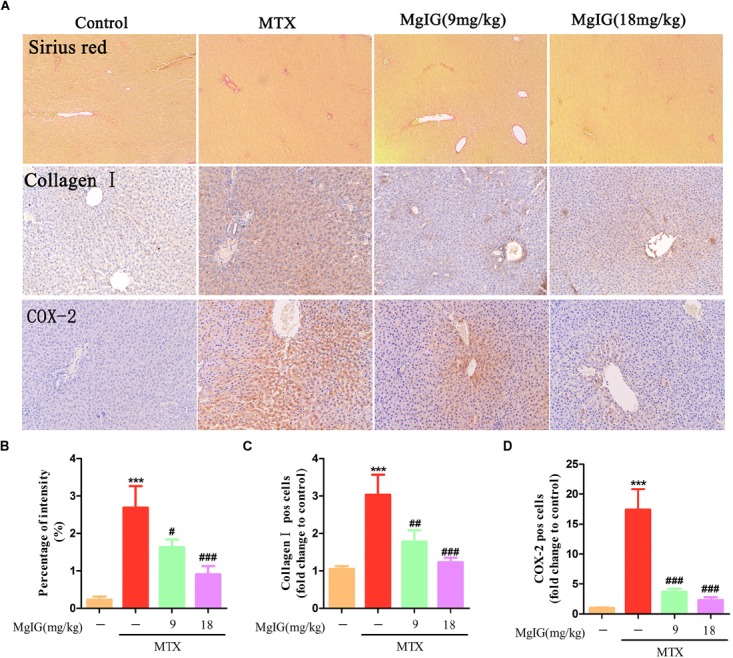
Effect of MgIG on MTX induced collagen deposition and expression of COX-2 in hepatic tissue. **(A)** Histological observation of collagen deposition by Sirius red staining and immunohistochemistry of collagen I and COX-2 in rat liver sections under light microscope, magnifications: × 200; **(B)** percentage (%) of intensity in Sirius red staining was calculated with the following formula: fibrosis area/whole area × 100; **(C,D)** quantification of percentage of collagen I and COX-2 positive cells (pos cells). The results are shown as means ±*SD* of three rats per group. Compared with control group: ^∗^*P* < 0.05, ^∗∗^*P* < 0.01, ^∗∗∗^*P* < 0.001; compared with MTX group: ^#^*P* < 0.05, ^##^*P* < 0.01, ^###^*P* < 0.001.

Sirius red staining exhibited that collagen deposition was aggravated in the MTX group compared to that in the control group (*P* < 0.001) (Figure 4A,B). Compared to the MTX group, collagen deposition was attenuated after administration with 9 and 18 mg/kg MgIG (*P* < 0.05 and *P* < 0.001, respectively). Moreover, the Immunohistochemical staining of collagen I showed that MgIG effectively relieved MTX-induced pathological changes of liver tissue, possibly being associated with fibrosis (Figure 4A,C).

### Hepatoprotective Effect of MgIG on MTX-Induced Hepatotoxicity May Be by Reducing COX-2

Decrease of COX-2 can effectively reverse MTX-induced liver damage (Ali et al., 2017). To investigate the relationship between COX-2 and the protective effects of MgIG on hepatotoxicity induced by MTX, we detected COX-2 expression in the liver by immunohistochemical assay. The MTX group had significantly higher COX-2 expression level in hepatic tissue than that of the control group (*P* < 0.001). After administration with 9 and 18 mg/kg MgIG for 7 days, the expression of COX-2 significantly reduced compared to that of the MTX group (*P* < 0.05 and *P* < 0.001, respectively) (Figure 4A,D and Supplementary Table S4).

### Effect of MgIG on MTX-Induced Intestinal Damage

**FIGURE 5:**
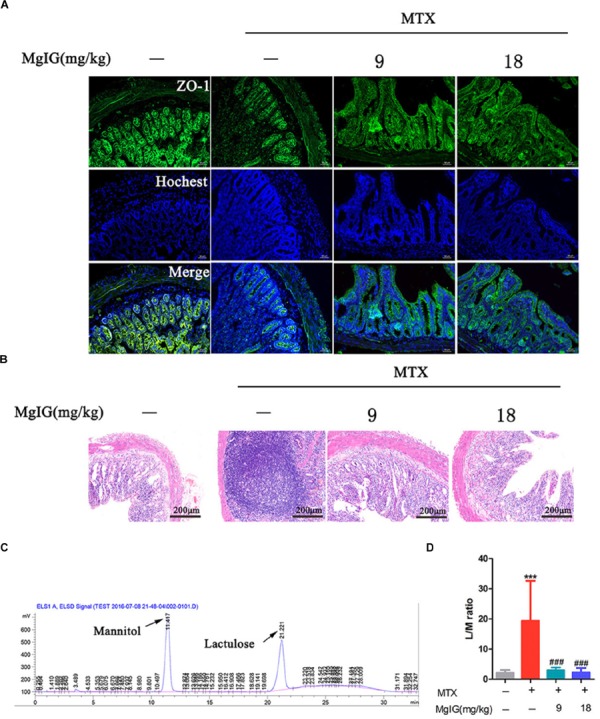
Effect of MgIG on MTX induced intestinal damage **(A)** Immunofluorescence analysis of ZO-1 (green); **(B)** intestinal sections were stained with HE; **(C)** chromatograms of lactose and mannitol; **(D)** lactulose and mannitol excretion in the urine of rats. The results are shown as means ±*SD* of six rats per group. Compared with control group: ^∗^*P* < 0.05, ^∗∗^*P* < 0.01, ^∗∗∗^*P* < 0.001; compared with MTX group: ^#^*P* < 0.05, ^##^*P* < 0.01, ^###^*P* < 0.001.

The MTX group underwent severe diarrhea. Administration of MTX significantly increased lactulose/mannitol ratio (L/M ratio) compared to that of the control group (^∗∗∗^*P* < 0.001). In contrast, the ratio significantly decreased after administration with 9 and 18 mg/kg MgIG compared to that of the MTX group (###*P* < 0.001 and ###*P* < 0.001, respectively) (Figure 5C,D and Supplementary Table S5). The regression equations of mannitol and lactose were *Y* = 3219X – 191.75 (*r*^2^ = 0.9978) and *Y* = 2812.2X – 175.05 (*r*^2^ = 0.9966), respectively. Zonula occludens-1 (ZO-1) is a key protein that maintains tight junctions in the intestinal tract. Immunofluorescence assay showed that MTX reduced the expression level of ZO-1 protein in intestinal tissue. However, the levels of 9 and 18 mg/kg MgIG groups were significantly higher than those of the MTX group (Figure 5A). In addition, there were inflammatory cell infiltration and crypt loss in the MTX group. Compared with the MTX group, the histopathological lesions were mitigated after pretreatment with 9 or 18 mg/kg MgIG (Figure 5B and Supplementary Data Sheet S3).

### MgIG Relieved MTX-Induced Intestinal Damage May Be by Reducing COX-2 and Colocalization of ZO-1 and Connexin 43 (Cx43)

**FIGURE 6:**
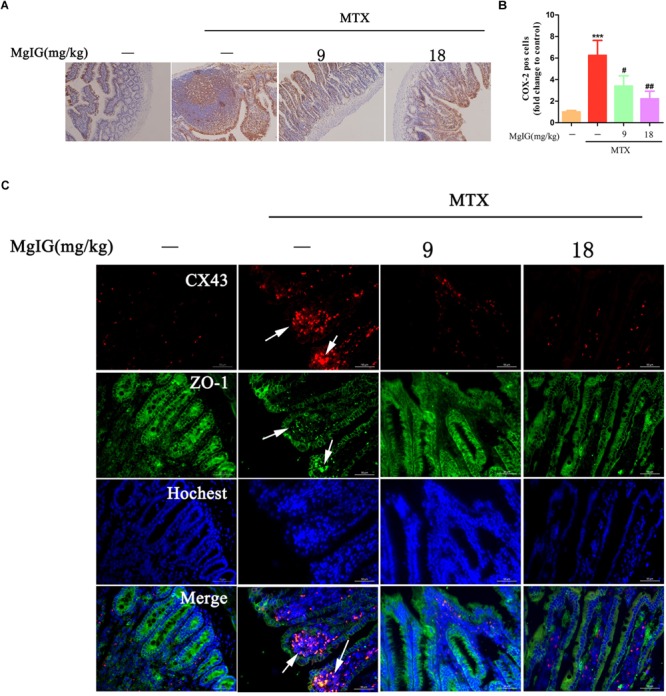
Hepatoprotective effect of MgIG on MTX-induced intestinal toxicity by regulating COX-2 and colocalization of ZO-1 and Cx43: **(A)** Effect of MgIG on MTX induced damage in intestinal tissue and immunohistochemistry of COX-2 in rat intestinal sections (Magnifications: × 200); **(B)** quantification of percentage of COX-2 positive cells (pos cells); **(C)** immunofluorescence analysis of Cx43 (red) and ZO-1 (green). The results are shown as means ±*SD* of three rats per group. Compared with control group: ^∗^*P* < 0.05, ^∗∗^*P* < 0.01, ^∗∗∗^*P* < 0.001; compared with MTX group: ^#^*P* < 0.05, ^##^*P* < 0.01, ^###^*P* < 0.001. The arrow means colocalization of ZO-1 and connexin 43 in liver.

COX-2 predominantly regulates colorectal inflammation and participates in MTX-induced intestinal injury (Natarajan et al., 2018). In this study, the MTX group had significantly higher COX-2 expression in intestinal tissue than that of the control group (^∗∗∗^*P* < 0.001). After administration with 9 and 18 mg/kg MgIG for seven days, the expressions of COX-2 significantly decreased compared to that of the MTX group (#*P* < 0.05 and ##*P* < 0.01, respectively) (Figure 6A,B, Supplementary Table S6, and Supplementary Data Sheet S4).

Cx43 protein is abnormally expressed in the case of inflammation-induced intestinal epithelial cell injury (Chotikatum et al., 2018). Herein, Cx43 was colocalized with ZO-1 at the top of intestinal villi after MTX treatment, with enhanced fluorescence. However, the colocalization of Cx43 and ZO-1 was barely observed after MgIG intervention (Figure 6C). Hence, MTX induced intestinal injury probably by affecting the localization of Cx43 and ZO-1, which may be restored by MgIG through the colocalization of them.

## Discussion

Hepatotoxicity results from various pathogenic factors (e.g., physical, chemical, and biological factors), leading to liver cell degeneration, necrosis and liver function changes (Wilke et al., 2007). MTX has been used to treat various cancers and inflammatory diseases (Bleyer, 1978). Nevertheless, hepatotoxicity is the most serious side effect of long-term MTX treatment, which is related to the accumulation of 7-hydroxymethotrexate, the main metabolite (West, 1997). Moreover, the hepatotoxicity and intestinal toxicity of MTX seriously affect the clinical therapeutic effects of MTX, thus requiring effective therapies. In this study, the effects of MgIG on MTX-induced hepatotoxicity and intestinal toxicity in rats were evaluated.

After MTX administration, rats underwent significant weight loss and increase of liver weight/body weight ratio, which were significantly restored by pretreatment with MgIG or GSH. Injecting MTX into the tail caused severe liver injury, manifested as augmented activities of ALT and AST, hepatocyte apoptosis, collagen deposition, intestinal permeability and liver pathological changes which were significantly alleviated by administration with 9 and 18 mg/kg MgIG. In other words, the liver functions were ameliorated. It has been demonstrated that MTX increased MDA in the liver, which then cause morphological and functional changes (Khafaga and El-Sayed, 2018). In our study, pretreatment with MgIG significantly decreased MDA level, whereas increased SOD, GSH and GPx levels in the liver compared to those of the MTX group, indicating that the toxic effects of MTX were attenuated.

Apoptosis of cells is an important phenomenon of liver injury (Mohamed and Magdy, 2017), and MTX-induced liver injury is also accompanied by hepatocyte apoptosis (Ali et al., 2017). As mentioned above, MgIG exerted hepatoprotective effects by inhibiting MTX-induced apoptosis of liver cells. On the other hand, collagen is the main component of connective tissue, but the increase of collagen content or deposition in hepatic tissue dominantly induces hepatic fibrosis (Mak et al., 2012). MgIG can reduce collagen deposition and COX-2 expression in the liver. Collagen deposition is mainly responsible for liver fibrosis, and COX-2 can promote MTX-induced hepatic inflammatory response and activation of hepatic stellate cells, further accelerating the progression of liver fibrosis (Fayez et al., 2018). Additionally, MTX is often first used to control rheumatism by modifying the immune system, and commonly prescribed with other drugs, including NSAIDs such as aspirin (Fries et al., 1990). COX-2 is the target for aspirin to exert anti-inflammatory effects (Strand, 2007). Our results suggested that MgIG decreased the expression level of COX-2 in the liver and attenuated MTX-induced increase in the intestinal permeability. In addition, MgIG relieved intestinal inflammation and decreased the expression of COX-2 in intestinal tissue.

Intestinal damage is also one of the main toxic effects of MTX in clinical practice. It damages the gastrointestinal tract mucosa, thereby disrupting the intestinal barrier functionality and allowing bacterial translocation to the liver to induce infections (Wiest et al., 2014). The sugar absorption test is based on the oral administration of lactulose and mannitol that differentially cross the impaired intestinal barrier into the circulation, after which they are rapidly cleared into urine (Sequeira et al., 2015). In our study, administration of MgIG significantly decreased L/M ratio compared to that of the MTX group. In addition, MgIG increased MTX-induced expression of ZO-1, a key protein maintaining intestinal tight junctions, in intestinal tissue compared to that of the MTX group. Furthermore, MgIG weakened MTX-induced colocalization of ZO-1 and Cx43, also verifying the protective effects of MgIG on the intestinal tract. As an important binding partner of Cx43 (Giepmans and Moolenaar, 1998), ZO-1 interacts with Cx43 and binds the periphery of GJ plaques where newly synthesized channels accrue, possibly affecting cell cycle stage (Singh et al., 2005).

**FIGURE 7:**
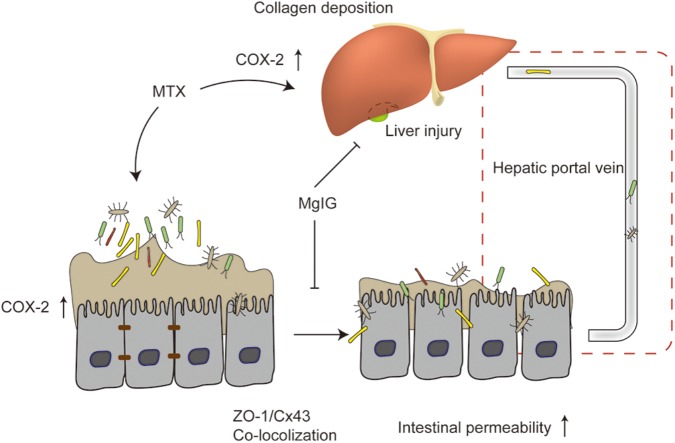
Mechanism of protective effect of MgIG on MTX-induced hepatotoxicity and intestinal damage: MgIG reduced MTX-induced abnormal expression of COX-2 in the liver and intestinal tissues, affecting collagen deposition in the liver and intestinal permeability. It also weakened MTX-induced colocalization of ZO-1 and Cx43. The increase in intestinal permeability was also essentially involved in MTX-induced liver injury. As evidenced by the inhibitory effects on MTX-induced intestinal damage, MgIG alleviated MTX-induced liver damage via multiple pathways.

At present, the protective effects of MgIG on liver injury are mainly attributed to STAT3 (Zhao Z. et al., 2017), hedgehog (Lu et al., 2017), NF-κB signaling pathway (Xu et al., 2016; Jiang et al., 2017a; Zhao X.J. et al., 2017) and phospholipase A2/arachidonic acid pathway (Xie et al., 2015). We herein, for the first time, proved that MgIG protected against MTX-induced hepatotoxicity and intestinal toxicity, and that it inhibited MTX-induced intestinal toxicity probably by regulating the expression of COX-2 and the colocalization of ZO-1 and Cx43. As suggested by the protective effects on MTX-induced intestinal damage, MgIG alleviated MTX-induced liver damage via multiple pathways (Figure 7).

Collectively, MgIG relieved not only MTX-induced increase in intestinal permeability, but also concomitant intestinal inflammation and COX-2 expression abnormalities. We postulated that MgIG protected against MTX-induced hepatotoxicity and intestinal damage by reducing abnormal liver and intestinal expressions of COX-2, as well as affecting collagen deposition in the liver and intestinal permeability. In summary, mitigating MTX-induced hepatotoxicity is of great significance to the improvement of life quality and treatment success rate. Given all histological and biochemical results, MgIG is a feasible drug for reducing the hepatic side effects of MTX during chemotherapy.

## Author Contributions

YL and YC conceived and designed the experiments. YC, HS, YX, and YFW performed the experiments. YC, JW, WC, and AW analyzed the data. ZS, XL, and YYW contributed reagents, materials, and analysis tools.

## Conflict of Interest Statement

The authors declare that the research was conducted in the absence of any commercial or financial relationships that could be construed as a potential conflict of interest.

## Supplementary Material

TABLE S1 Original data for body weight and liver weight/body weight ratio.

TABLE S2 Original data for serum and hepatic enzymatic levels.

TABLE S3 Original data for semi-quantitative analysis of TUNEL-positive cells.

TABLE S4 Original data for quantification of sirius red staining, collagen I and COX-2 positive cells in liver sections.

TABLE S5 Original data for lactulose and mannitol excretion.

TABLE S6 Original data for quantification of COX-2 positive cells.

DATA SHEET S1 Original data for liver histopathological lesions.

DATA SHEET S2 Original data for western blotting.

DATA SHEET S3 Original data for intestinal histopathological lesions.

DATA SHEET S4 Original data for immunohistochemistry of intestinal COX-2.

DATA SHEETS S5, S6 Standard biosecurity and institutional safety procedures.
