# Supplementary figures and images for: Protective Effects of Magnesium Glycyrrhizinate on Methotrexate-Induced Hepatotoxicity and Intestinal Toxicity May Be by Reducing COX-2
Source: Front Pharmacol. 2019 Mar 25;10:119. doi: 10.3389/fphar.2019.00119 (PMC6444054; doi:10.3389/fphar.2019.00119)

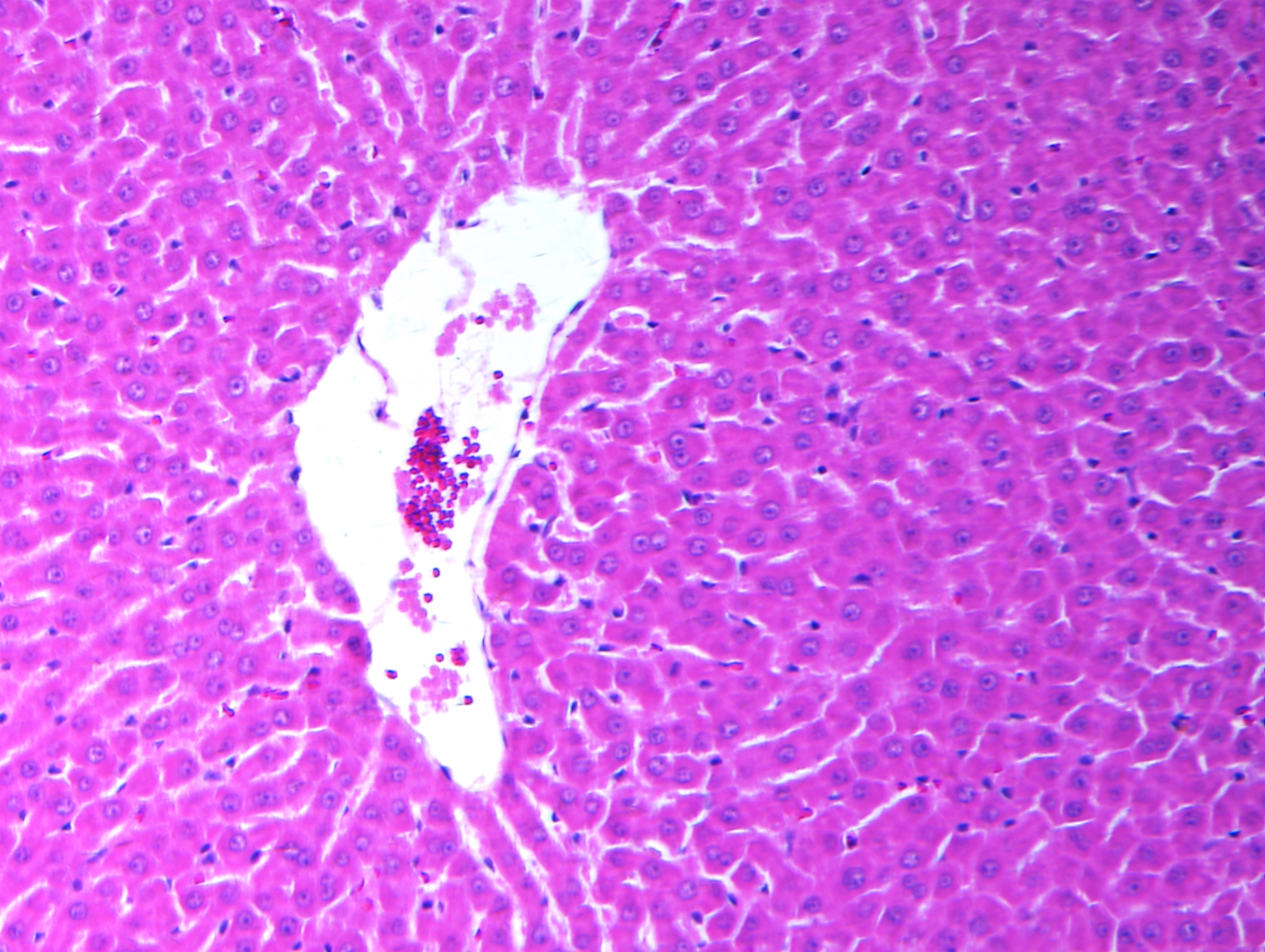

Supplement: DATA SHEET S1 — Original data for liver histopathological lesions. [file Data_Sheet_1.ZIP › FIG1D/GSH/GSH1.tif]

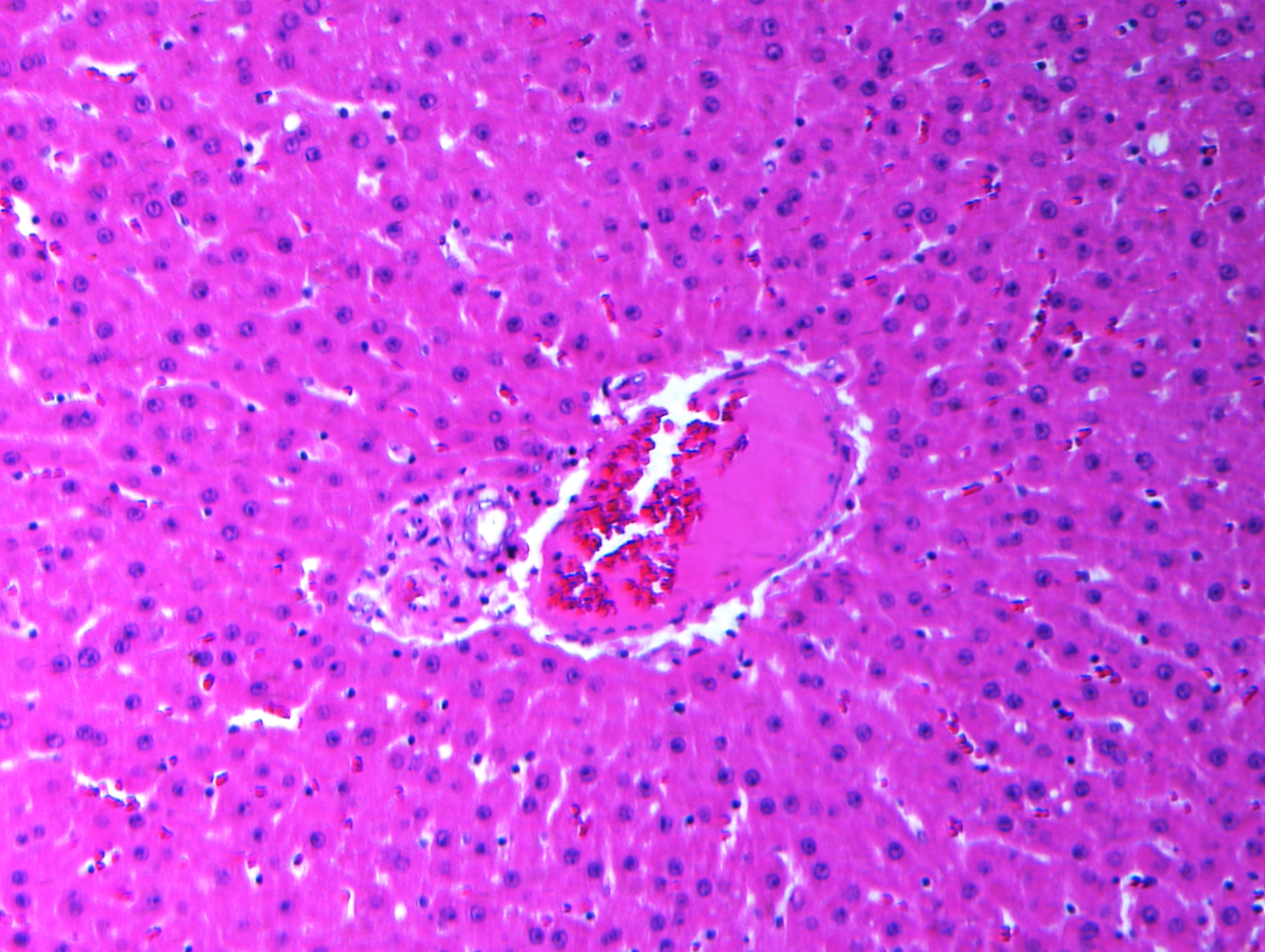

Supplement: DATA SHEET S1 — Original data for liver histopathological lesions. [file Data_Sheet_1.ZIP › FIG1D/MTX/MTX-1.tif]

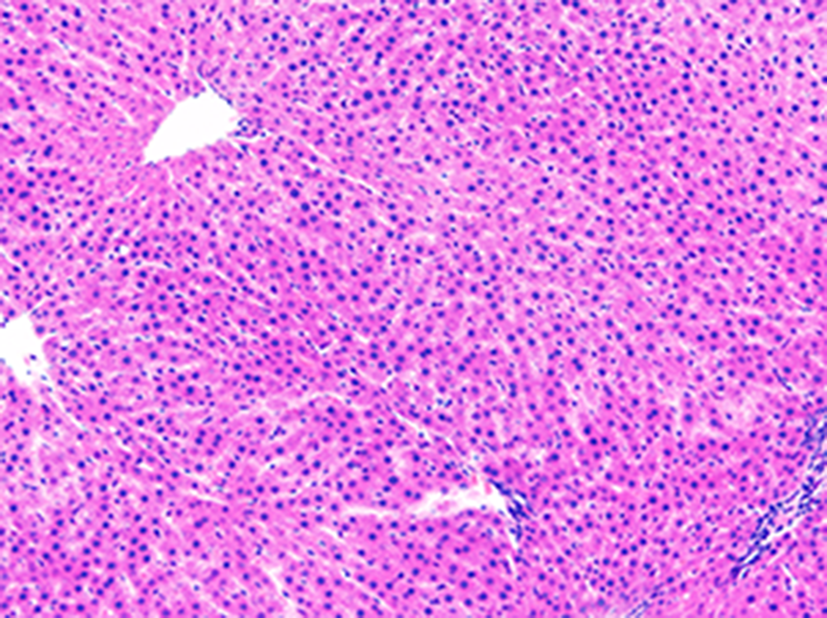

Supplement: DATA SHEET S1 — Original data for liver histopathological lesions. [file Data_Sheet_1.ZIP › FIG1D/MgIG(18mgkg)/MgIG18-6.tif]

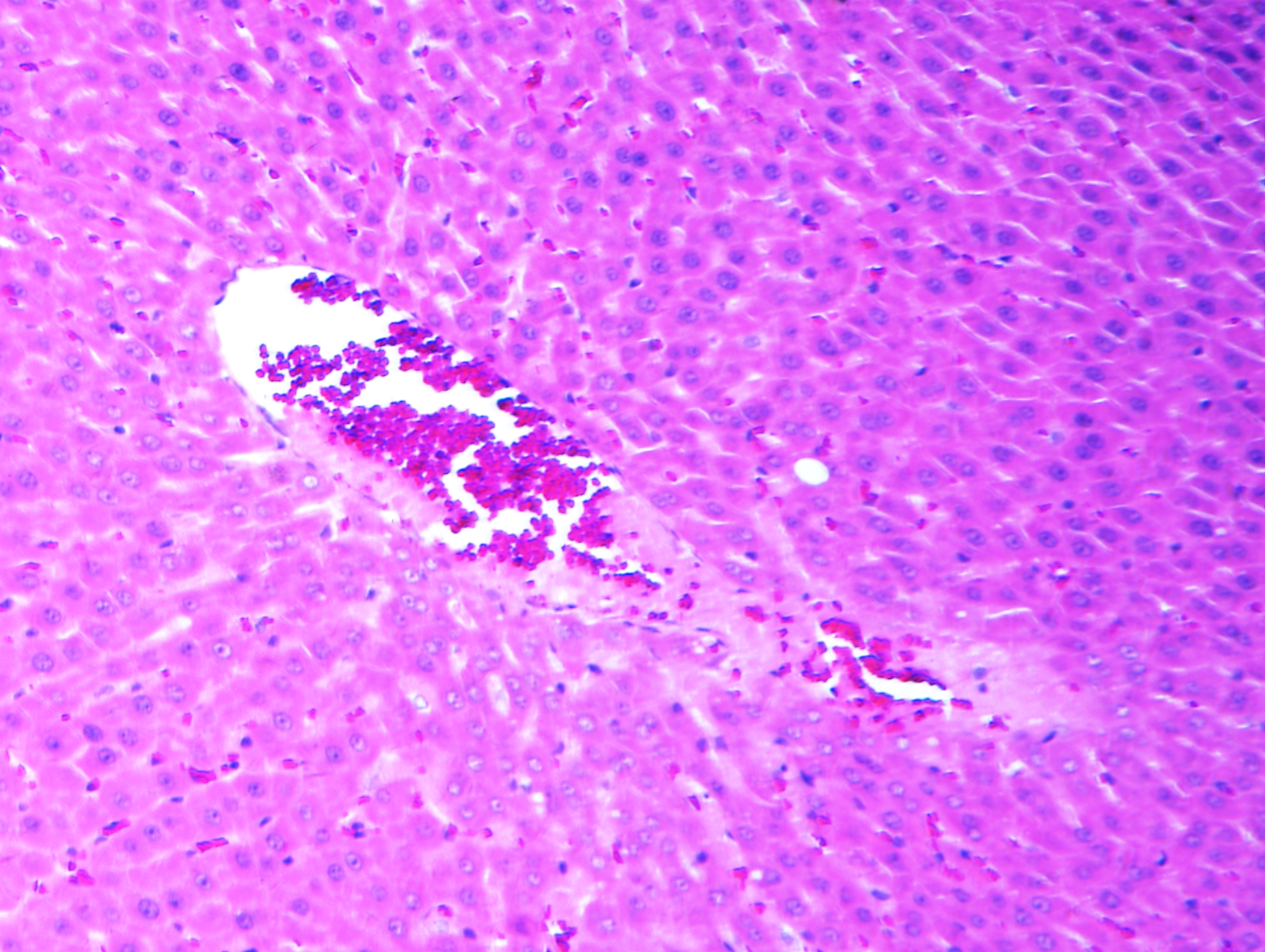

Supplement: DATA SHEET S1 — Original data for liver histopathological lesions. [file Data_Sheet_1.ZIP › FIG1D/MgIG(9mgkg)/MgIG9-1.tif]

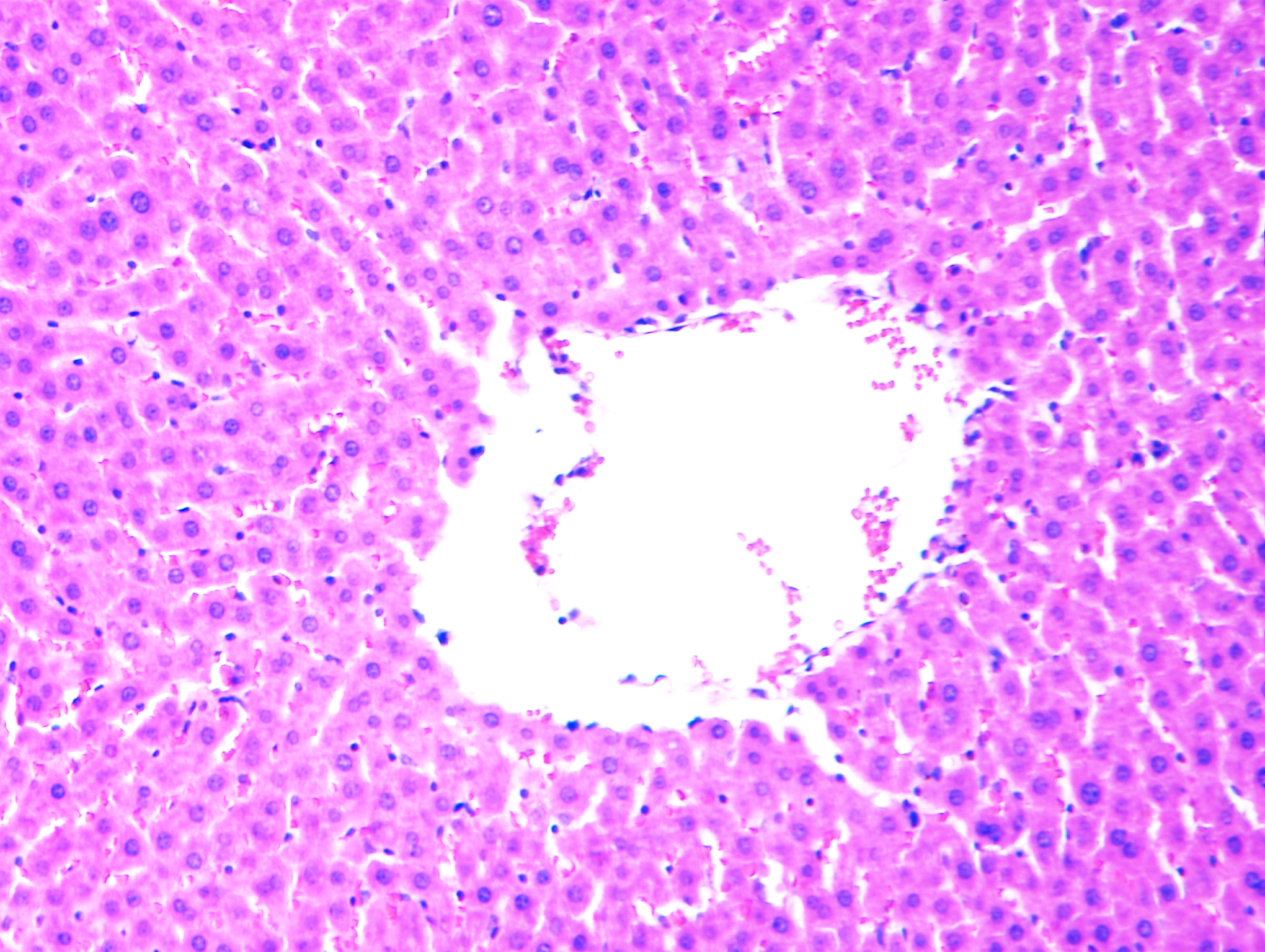

Supplement: DATA SHEET S1 — Original data for liver histopathological lesions. [file Data_Sheet_1.ZIP › FIG1D/control/Control-1.tif]

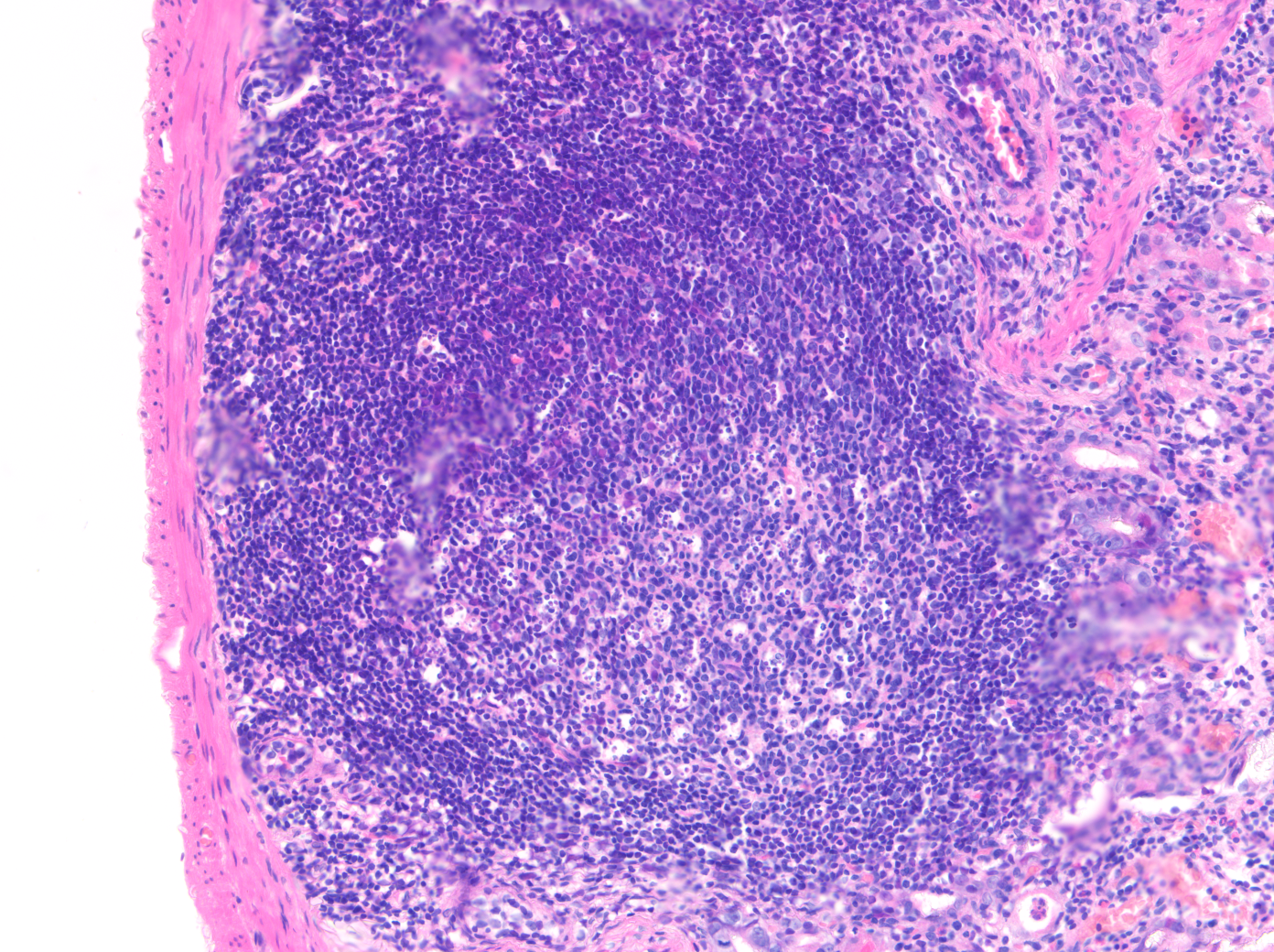

Supplement: DATA SHEET S3 — Original data for intestinal histopathological lesions. [file Data_Sheet_3.ZIP › FIG5B/MTX/MTX-1.tif]

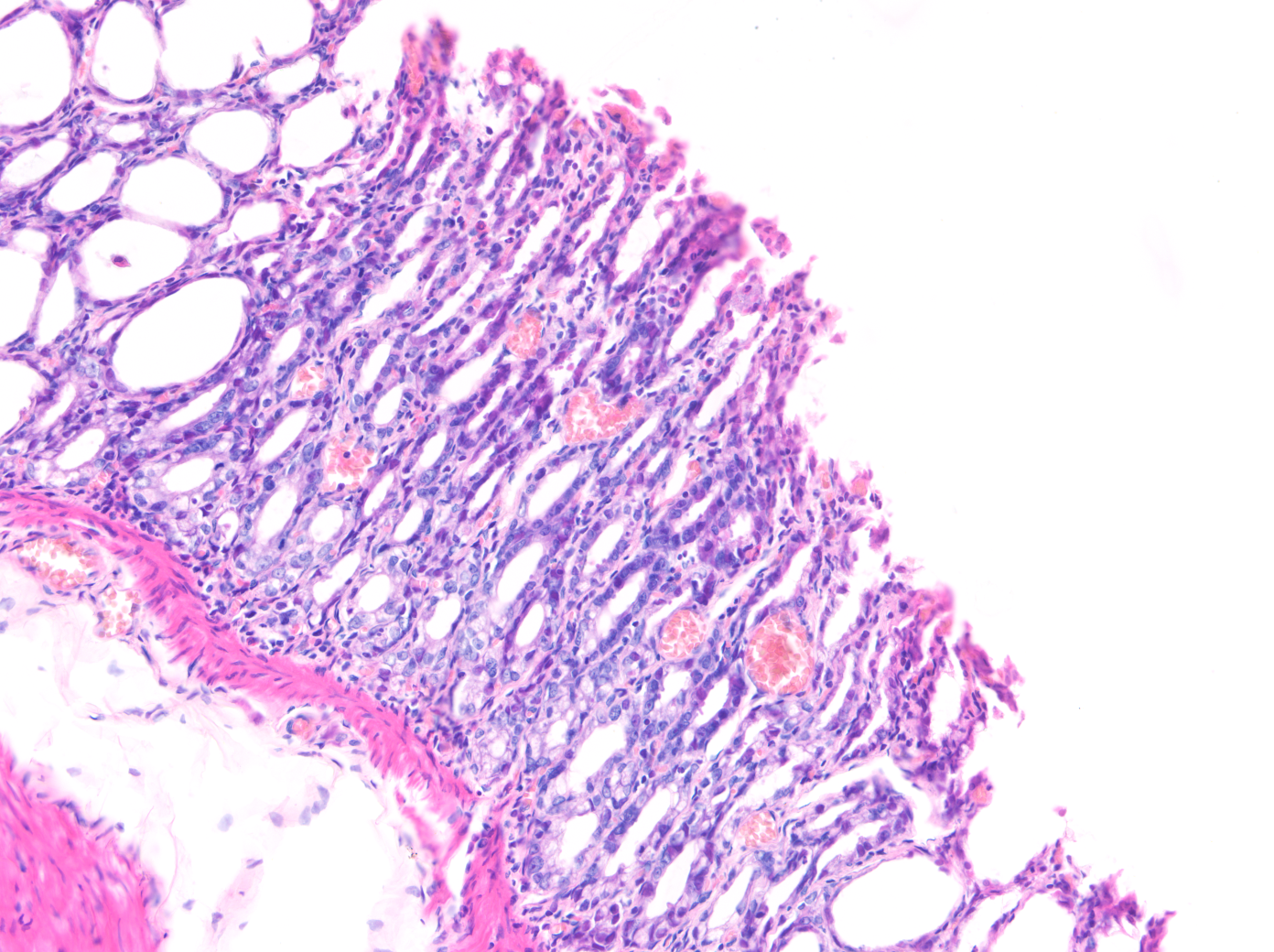

Supplement: DATA SHEET S3 — Original data for intestinal histopathological lesions. [file Data_Sheet_3.ZIP › FIG5B/MTX/MTX-2.tif]

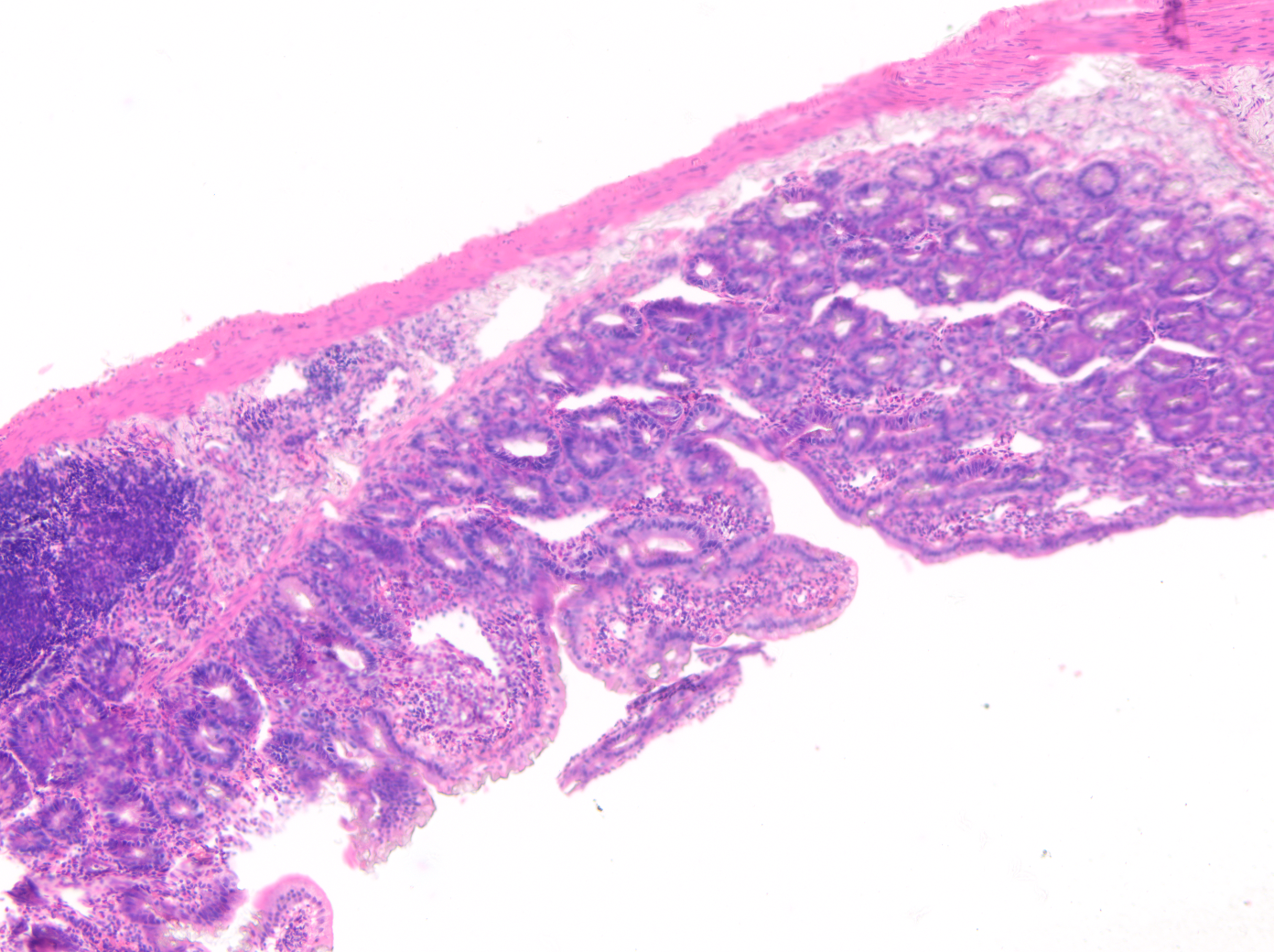

Supplement: DATA SHEET S3 — Original data for intestinal histopathological lesions. [file Data_Sheet_3.ZIP › FIG5B/MTX/MTX-3.tif]

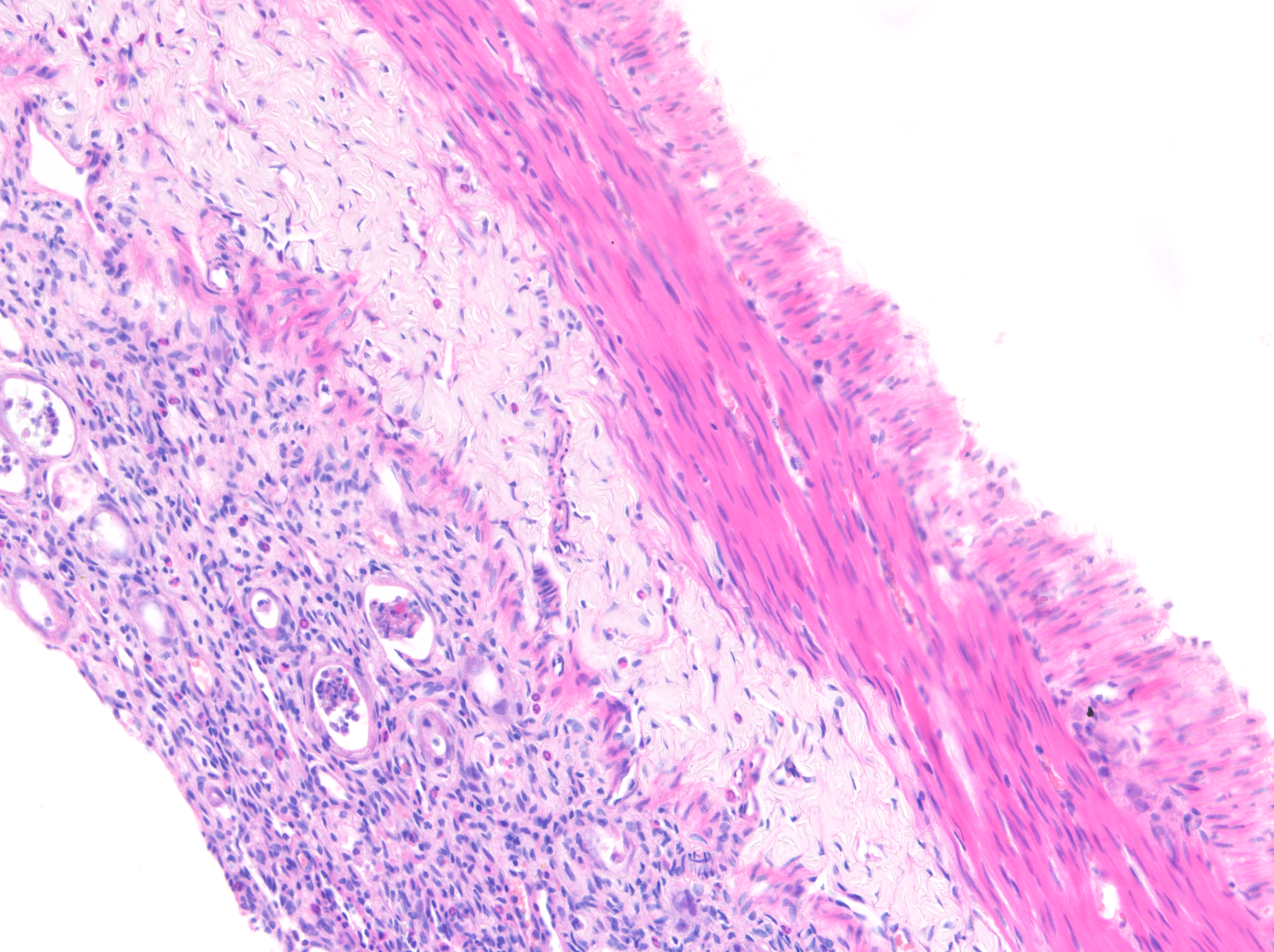

Supplement: DATA SHEET S3 — Original data for intestinal histopathological lesions. [file Data_Sheet_3.ZIP › FIG5B/MgIG(18mgkg)/MgIG18-1.tif]

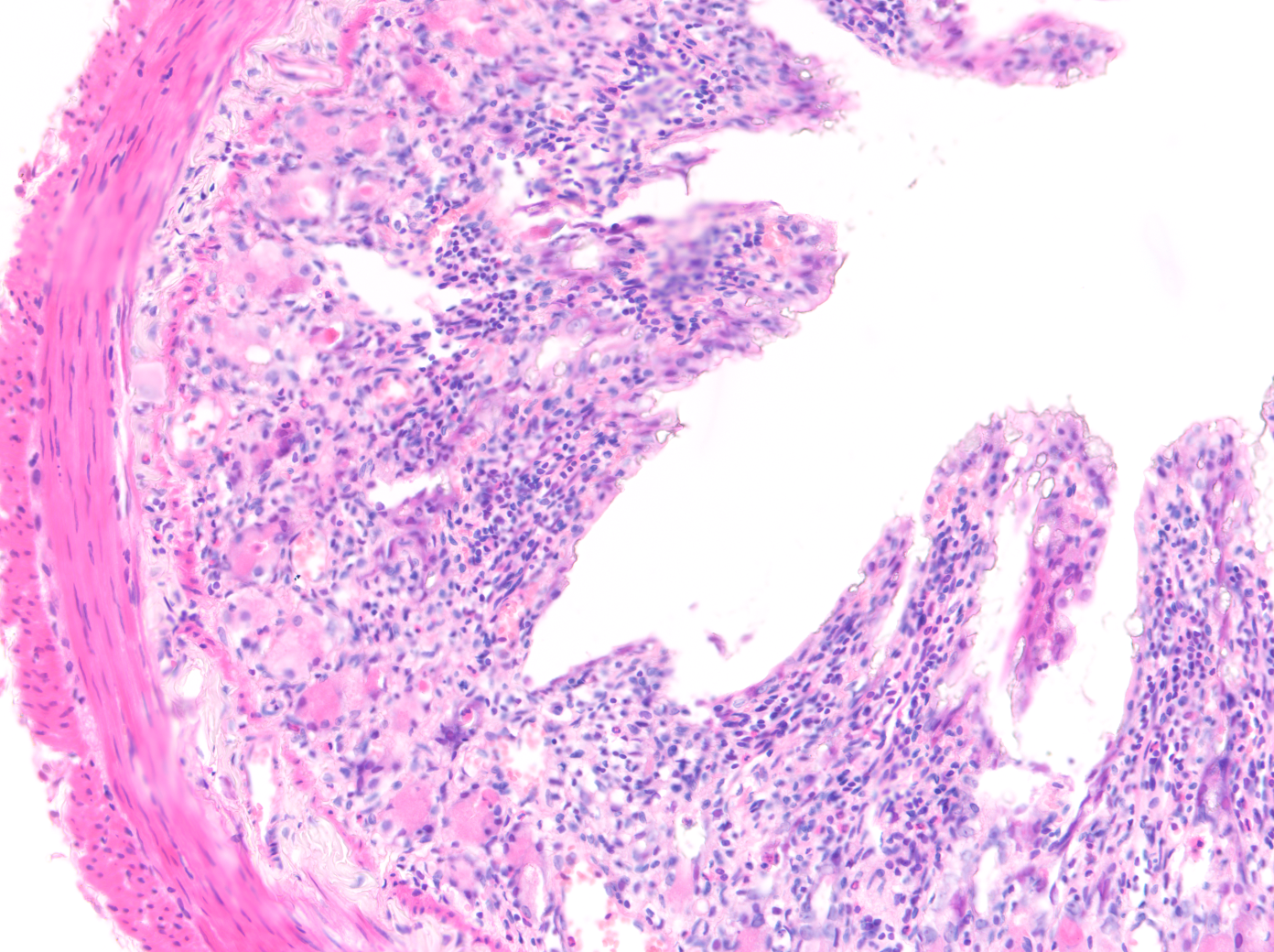

Supplement: DATA SHEET S3 — Original data for intestinal histopathological lesions. [file Data_Sheet_3.ZIP › FIG5B/MgIG(18mgkg)/MgIG18-2.tif]

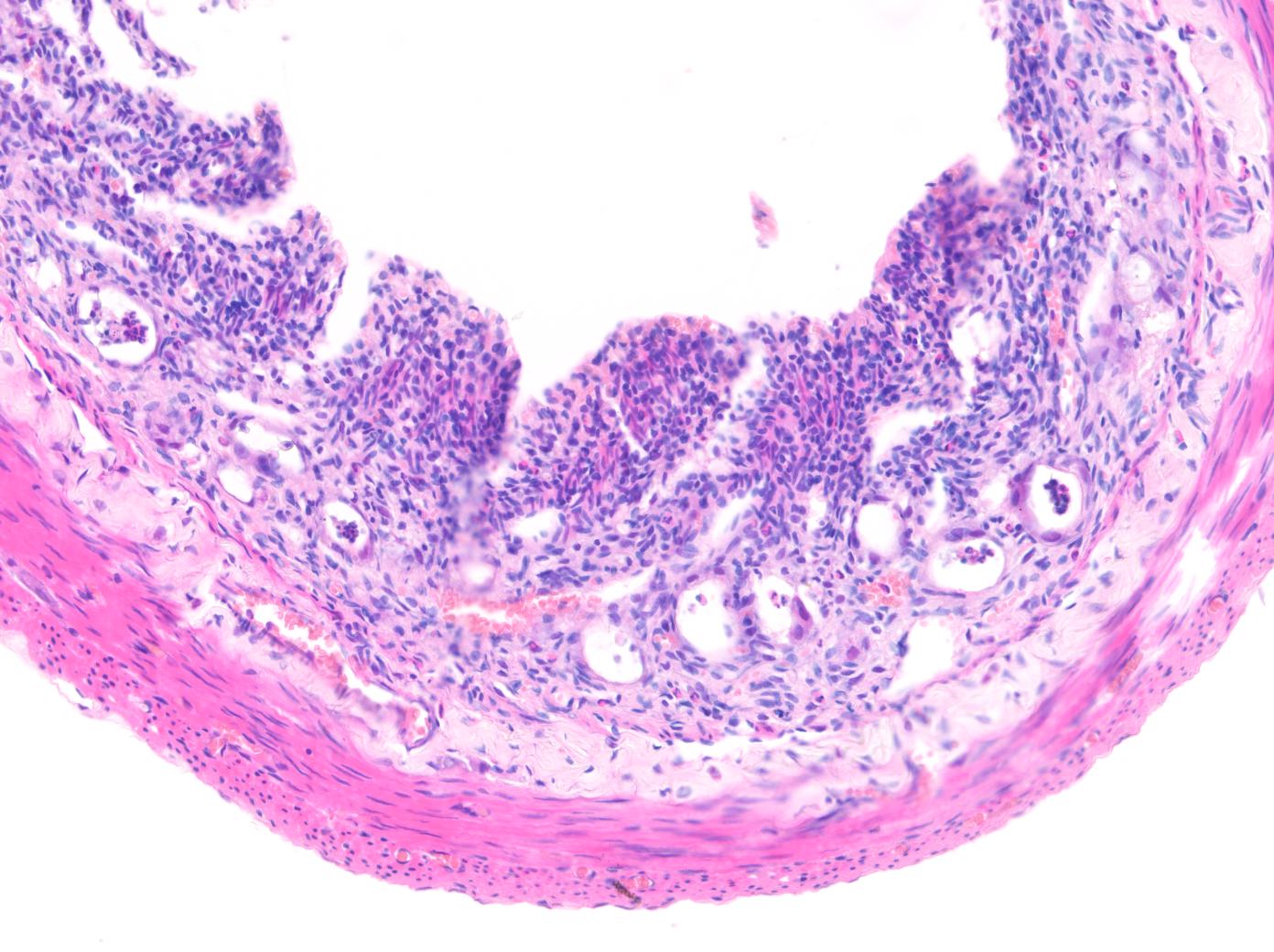

Supplement: DATA SHEET S3 — Original data for intestinal histopathological lesions. [file Data_Sheet_3.ZIP › FIG5B/MgIG(18mgkg)/MgIG18-3.tif]

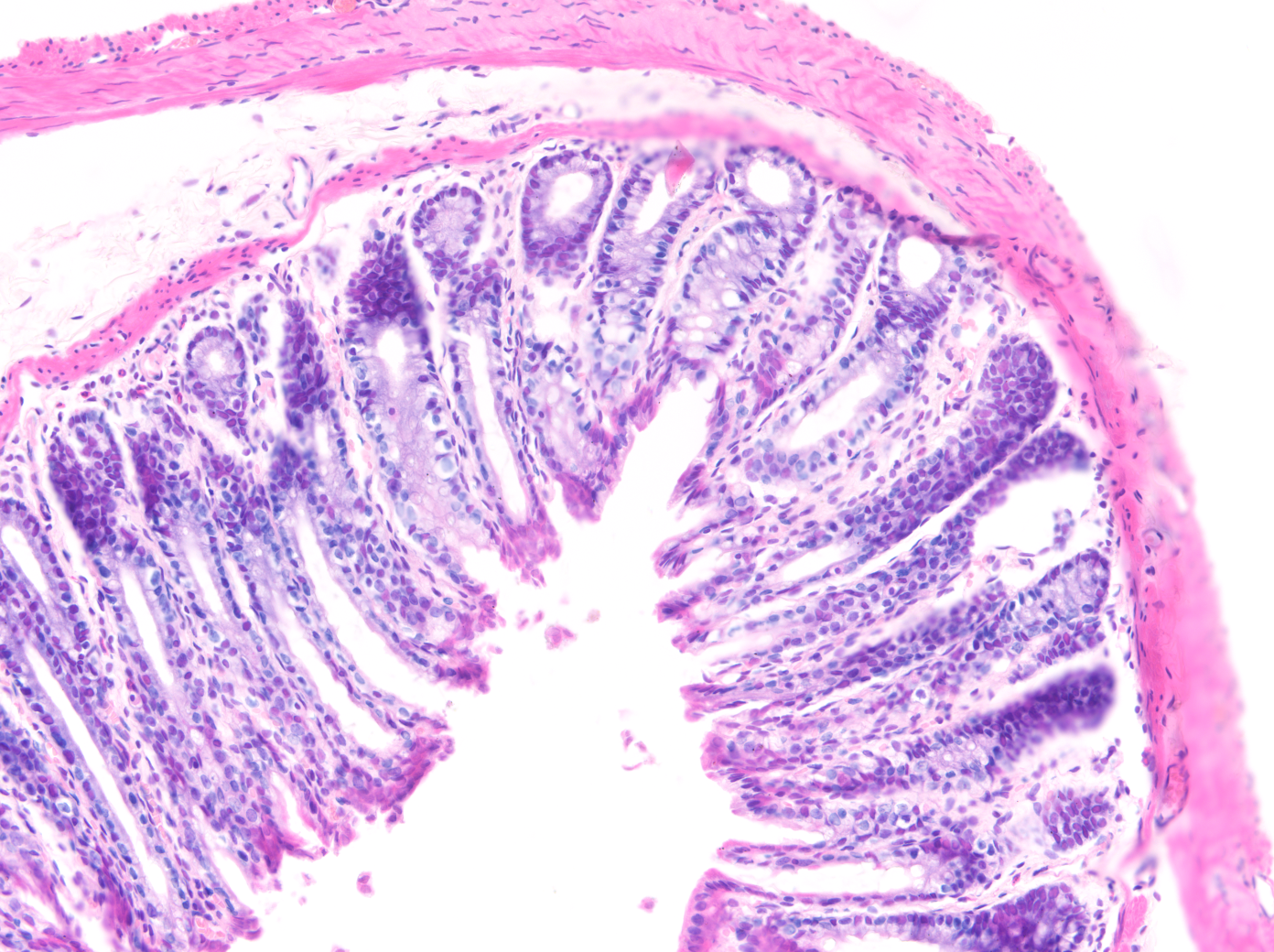

Supplement: DATA SHEET S3 — Original data for intestinal histopathological lesions. [file Data_Sheet_3.ZIP › FIG5B/MgIG(9mgkg)/MgIG9-1.tif]

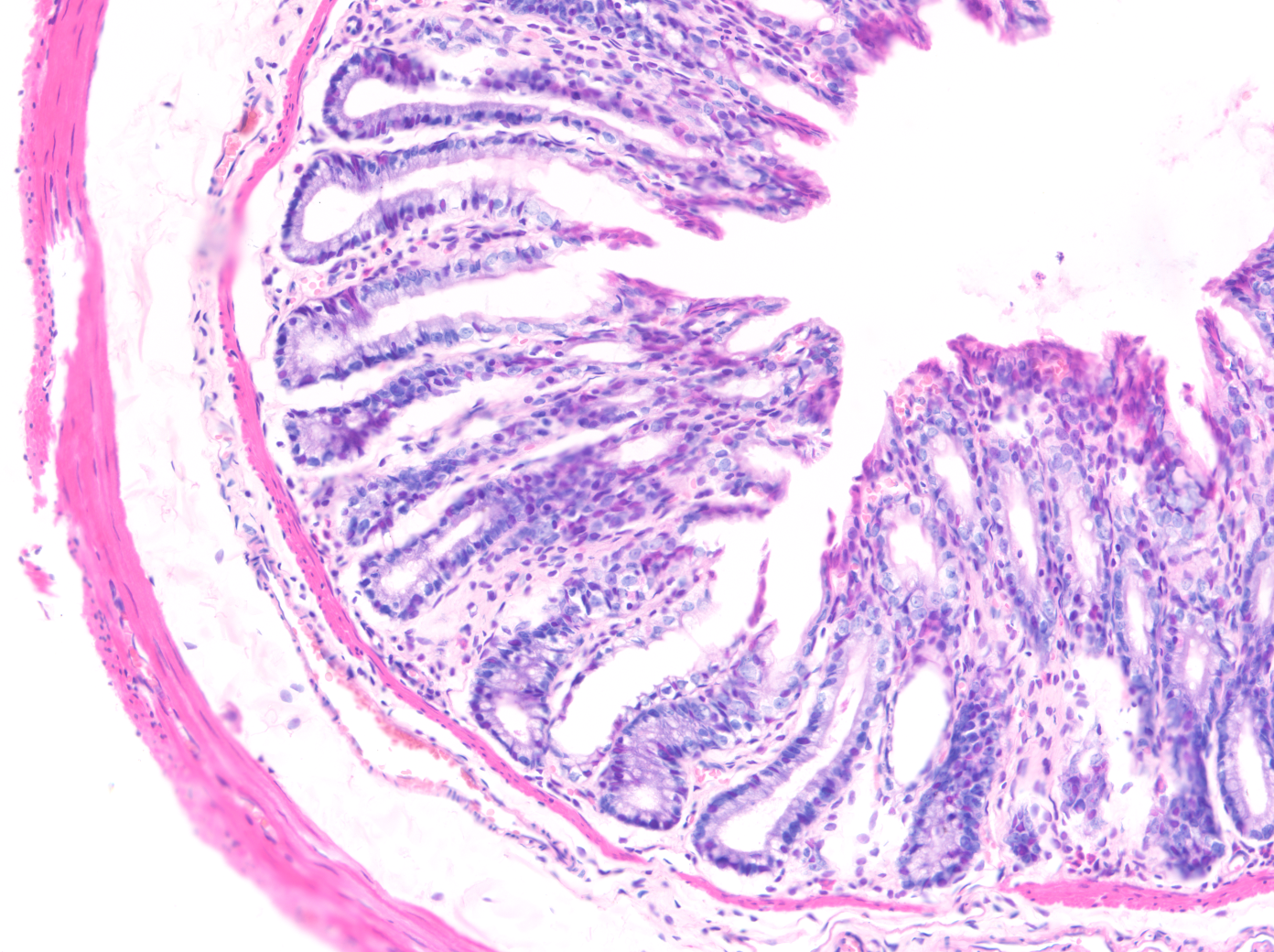

Supplement: DATA SHEET S3 — Original data for intestinal histopathological lesions. [file Data_Sheet_3.ZIP › FIG5B/MgIG(9mgkg)/MgIG9-2.tif]

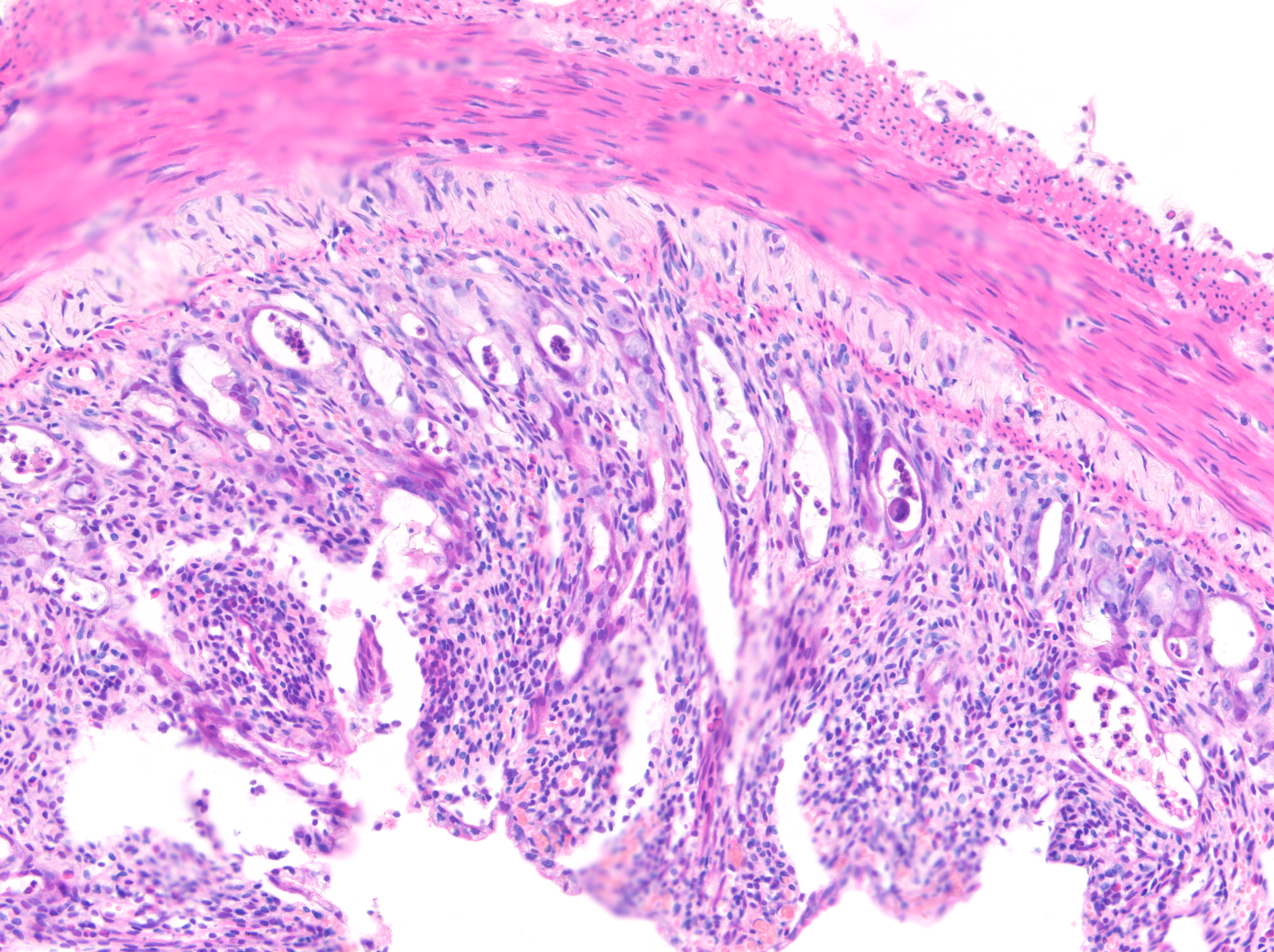

Supplement: DATA SHEET S3 — Original data for intestinal histopathological lesions. [file Data_Sheet_3.ZIP › FIG5B/MgIG(9mgkg)/MgIG9-3.tif]

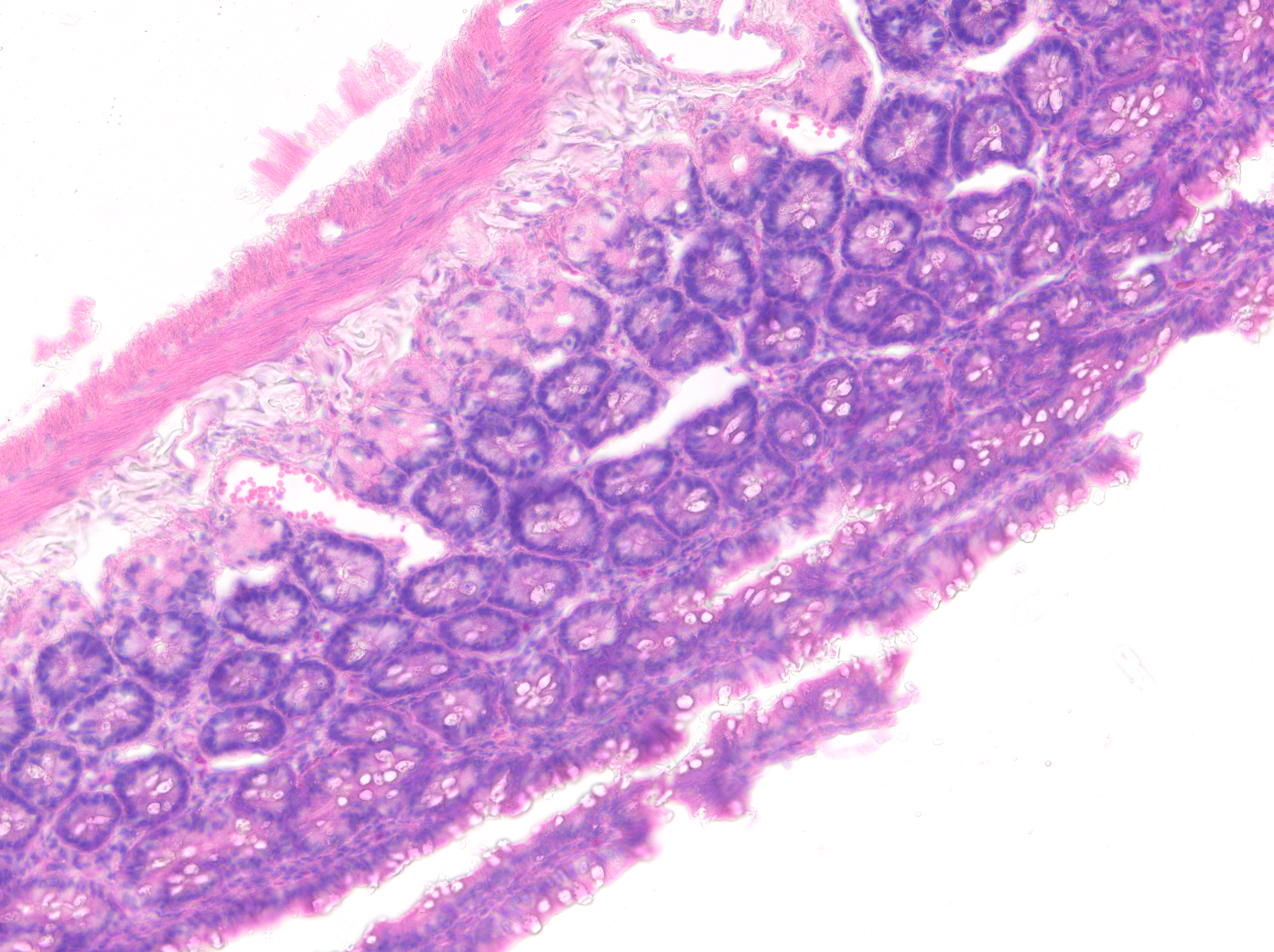

Supplement: DATA SHEET S3 — Original data for intestinal histopathological lesions. [file Data_Sheet_3.ZIP › FIG5B/control/control-1.tif]

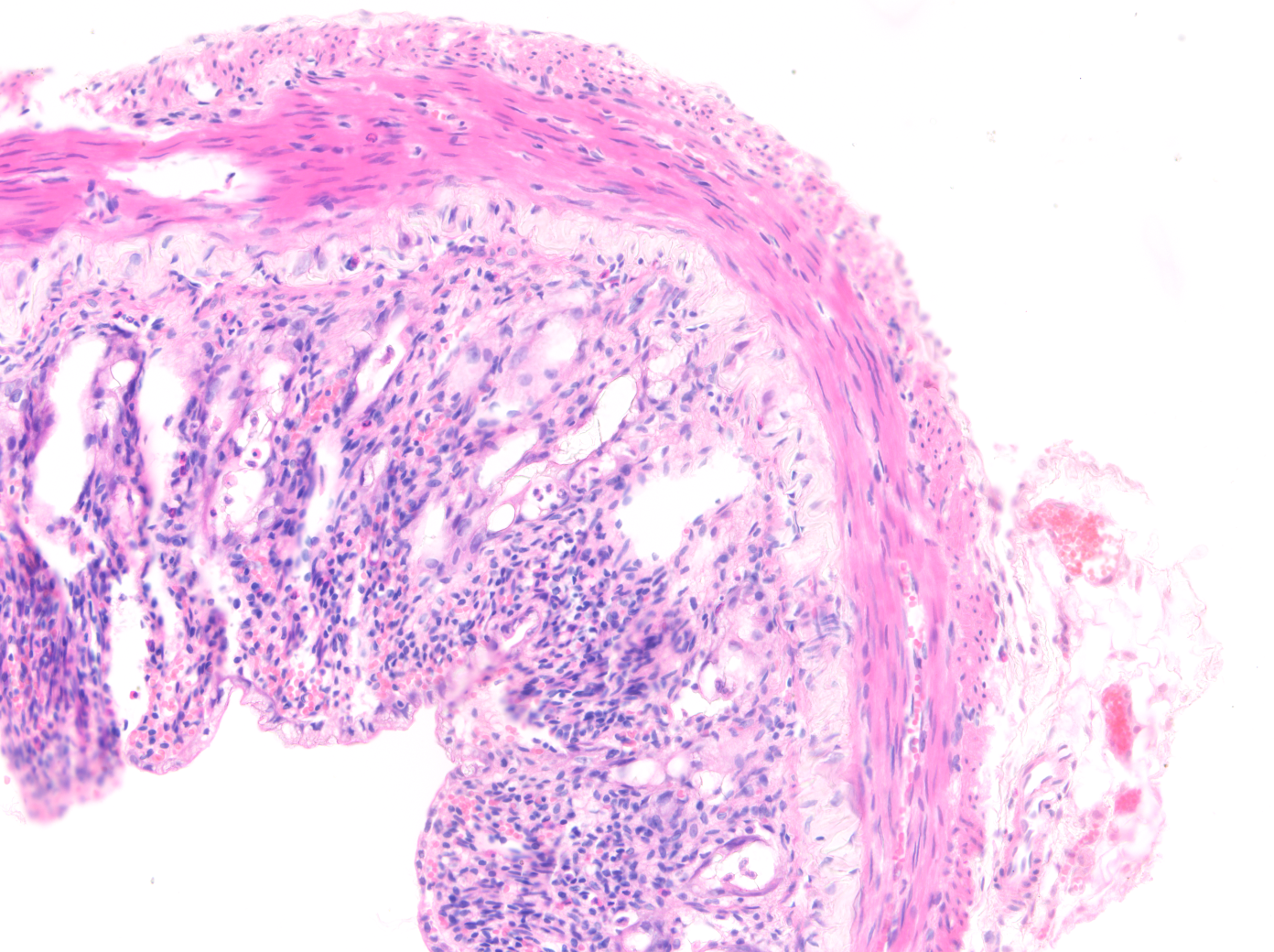

Supplement: DATA SHEET S3 — Original data for intestinal histopathological lesions. [file Data_Sheet_3.ZIP › FIG5B/control/control-2.tif]

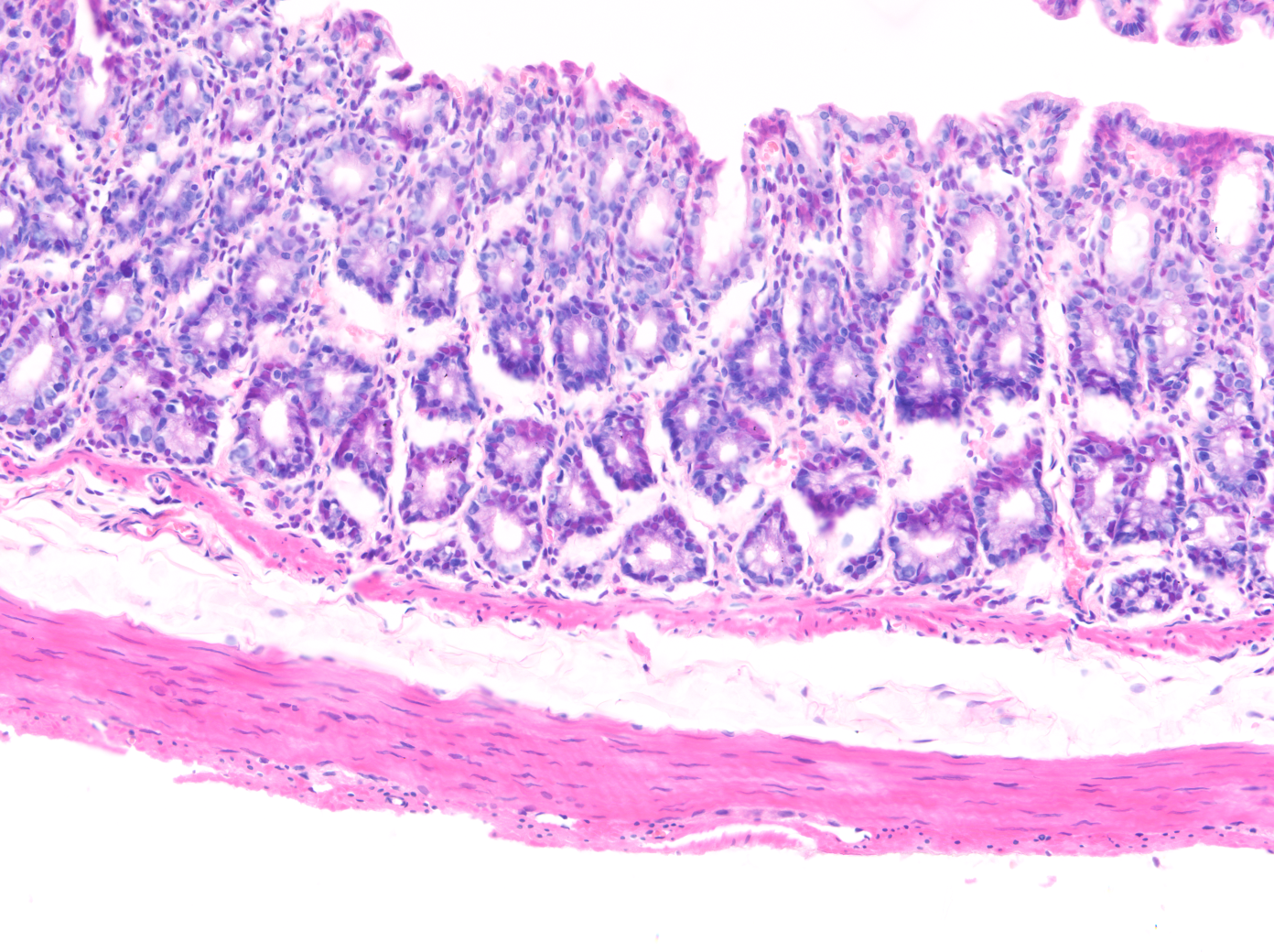

Supplement: DATA SHEET S3 — Original data for intestinal histopathological lesions. [file Data_Sheet_3.ZIP › FIG5B/control/control-3.tif]

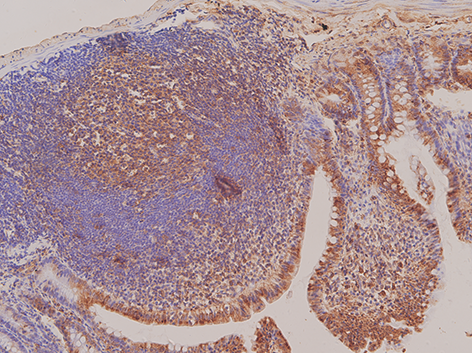

Supplement: DATA SHEET S4 — Original data for immunohistochemistry of intestinal COX-2. [file Data_Sheet_4.ZIP › FIG6A/COX-2/MTX/MTX-1.tif]

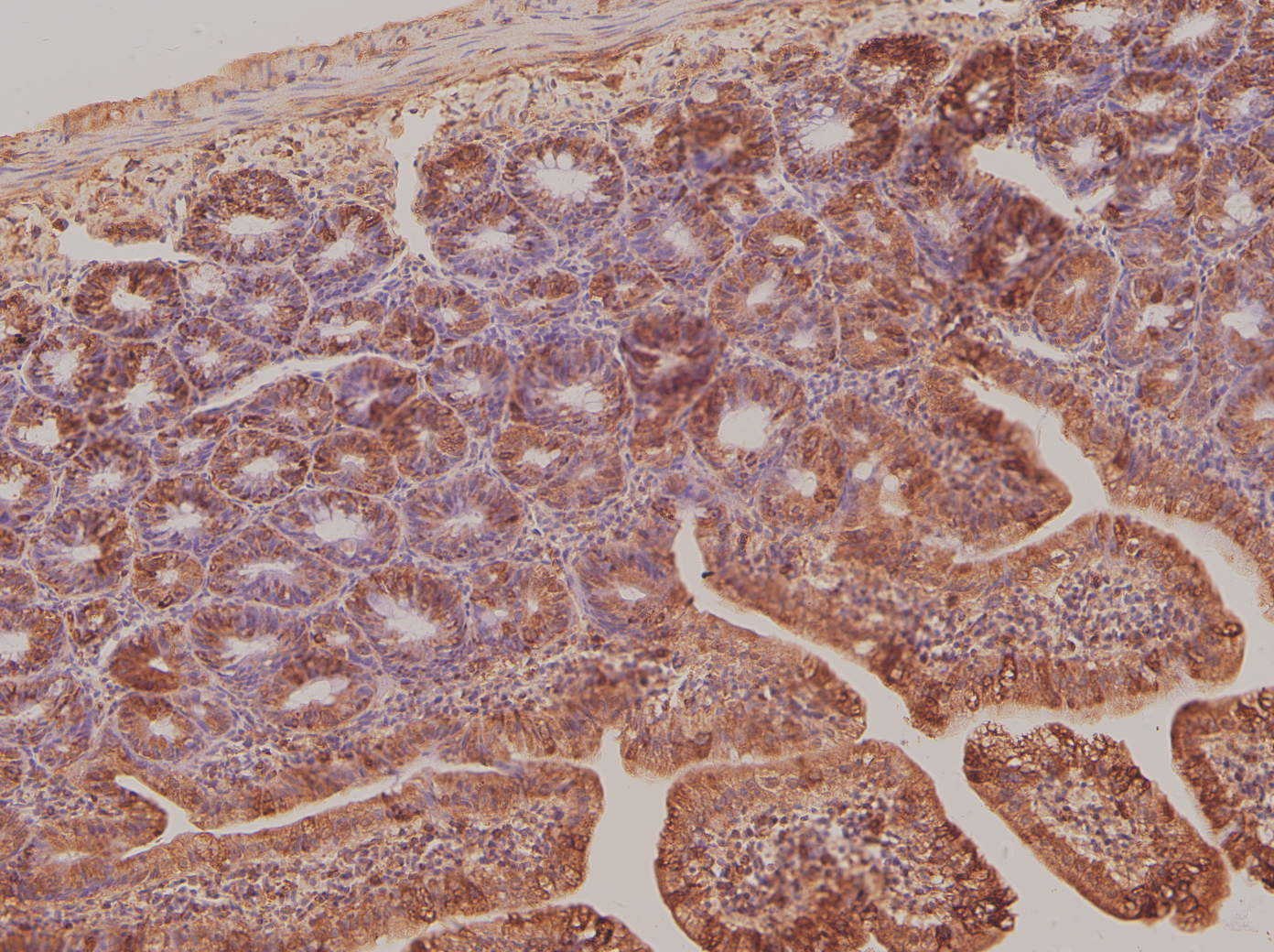

Supplement: DATA SHEET S4 — Original data for immunohistochemistry of intestinal COX-2. [file Data_Sheet_4.ZIP › FIG6A/COX-2/MTX/MTX-2.tif]

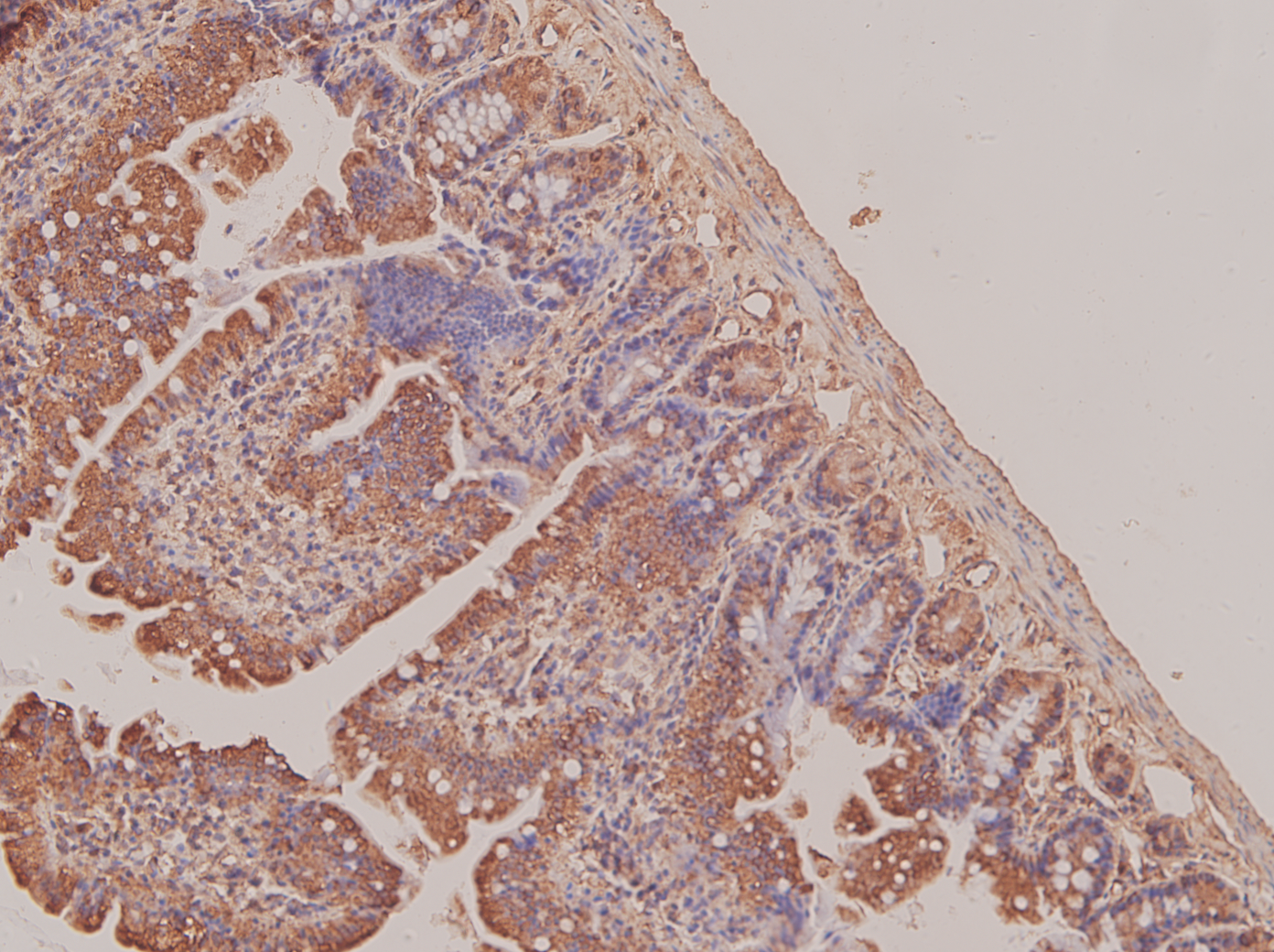

Supplement: DATA SHEET S4 — Original data for immunohistochemistry of intestinal COX-2. [file Data_Sheet_4.ZIP › FIG6A/COX-2/MTX/MTX-3.tif]

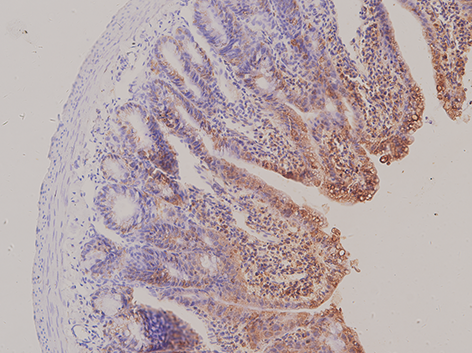

Supplement: DATA SHEET S4 — Original data for immunohistochemistry of intestinal COX-2. [file Data_Sheet_4.ZIP › FIG6A/COX-2/MgIG18/MgIG18-1.tif]

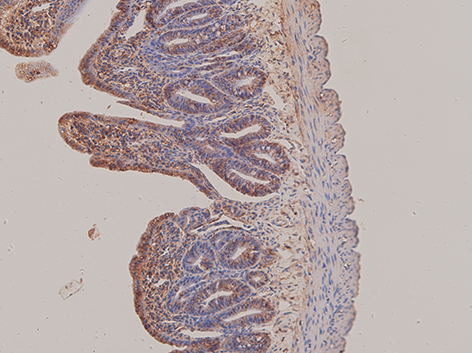

Supplement: DATA SHEET S4 — Original data for immunohistochemistry of intestinal COX-2. [file Data_Sheet_4.ZIP › FIG6A/COX-2/MgIG18/MgIG18-2.tif]

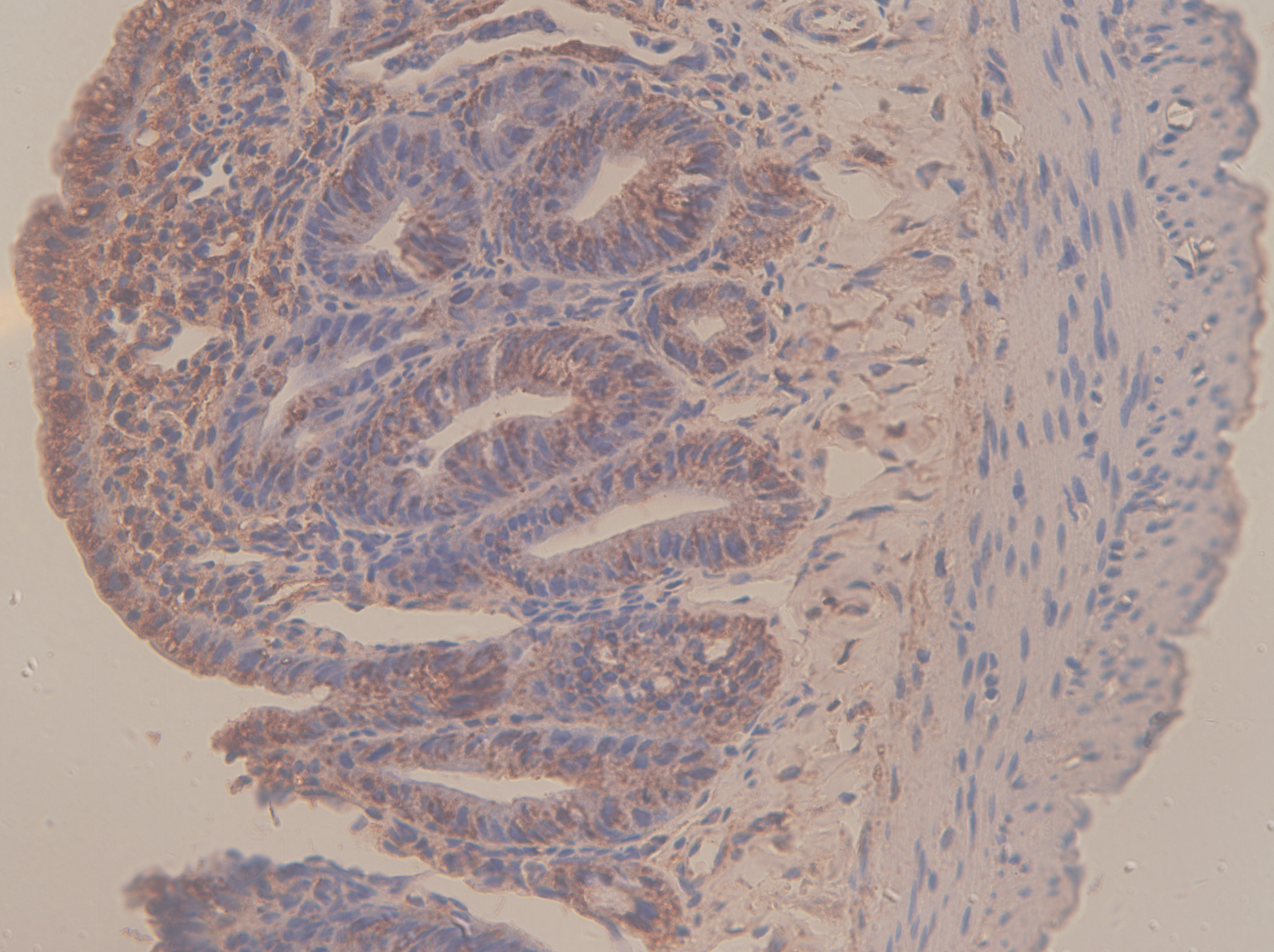

Supplement: DATA SHEET S4 — Original data for immunohistochemistry of intestinal COX-2. [file Data_Sheet_4.ZIP › FIG6A/COX-2/MgIG18/MgIG18-3.tif]

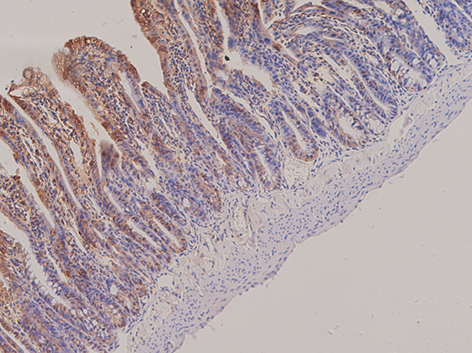

Supplement: DATA SHEET S4 — Original data for immunohistochemistry of intestinal COX-2. [file Data_Sheet_4.ZIP › FIG6A/COX-2/MgIG9/MgIG9-1.tif]

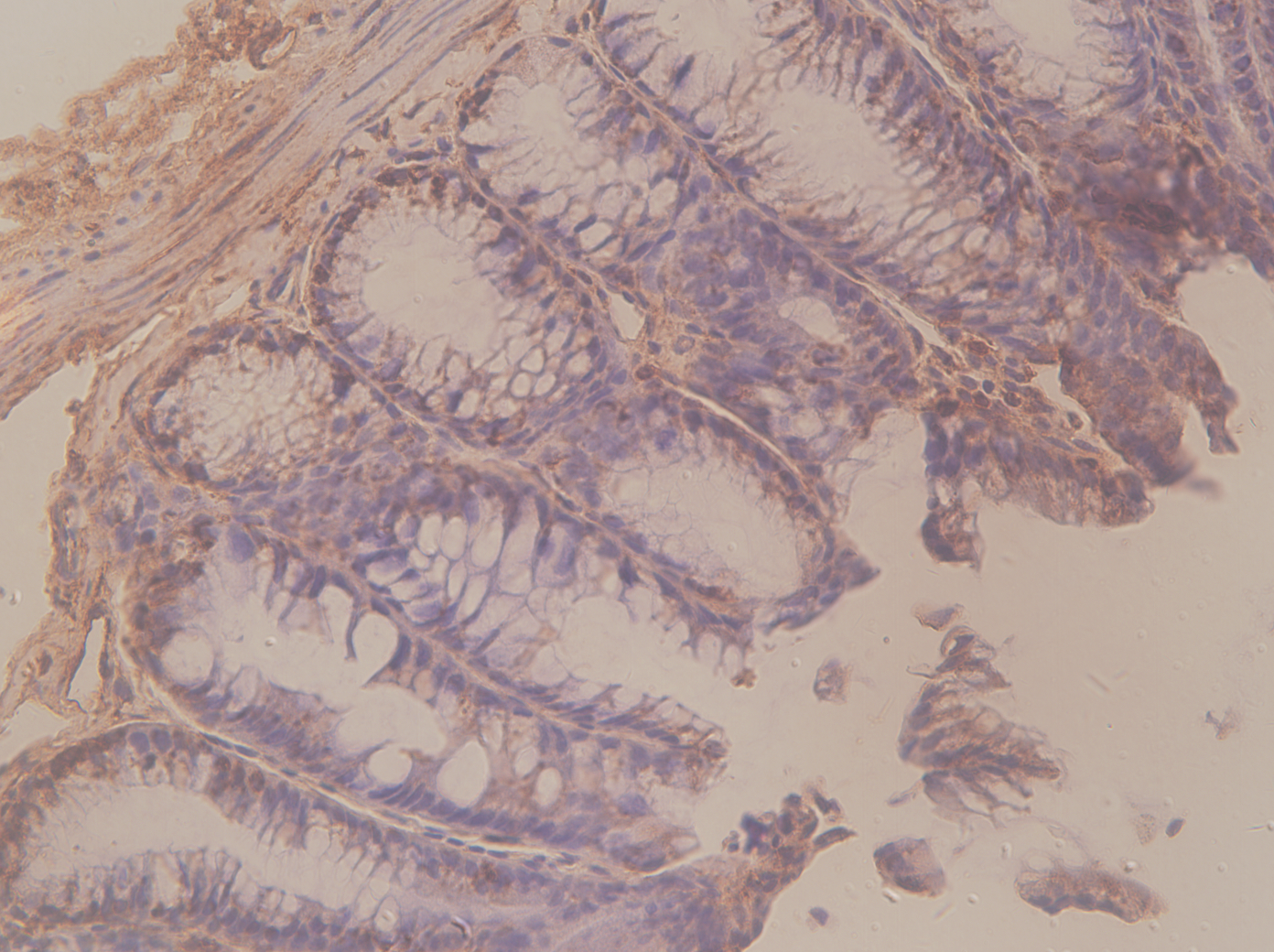

Supplement: DATA SHEET S4 — Original data for immunohistochemistry of intestinal COX-2. [file Data_Sheet_4.ZIP › FIG6A/COX-2/MgIG9/MgIG9-2.tif]

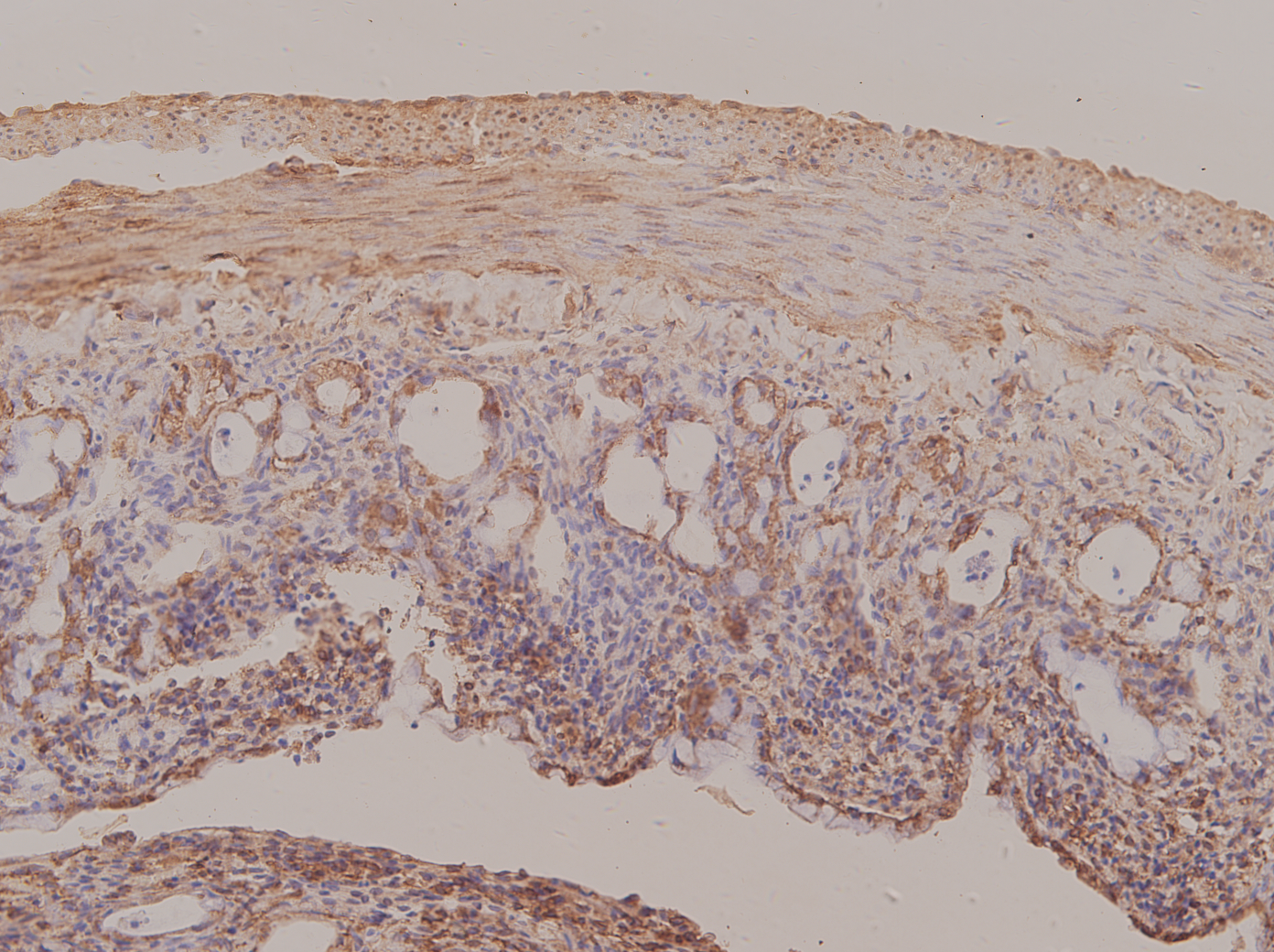

Supplement: DATA SHEET S4 — Original data for immunohistochemistry of intestinal COX-2. [file Data_Sheet_4.ZIP › FIG6A/COX-2/MgIG9/MgIG9-3.tif]

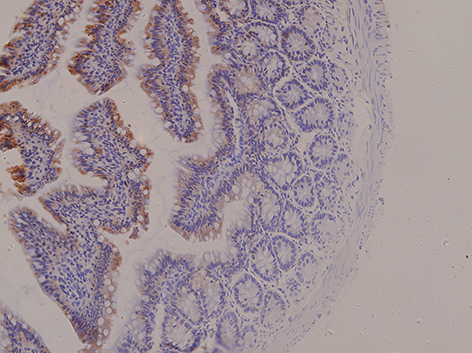

Supplement: DATA SHEET S4 — Original data for immunohistochemistry of intestinal COX-2. [file Data_Sheet_4.ZIP › FIG6A/COX-2/control/control-1.tif]

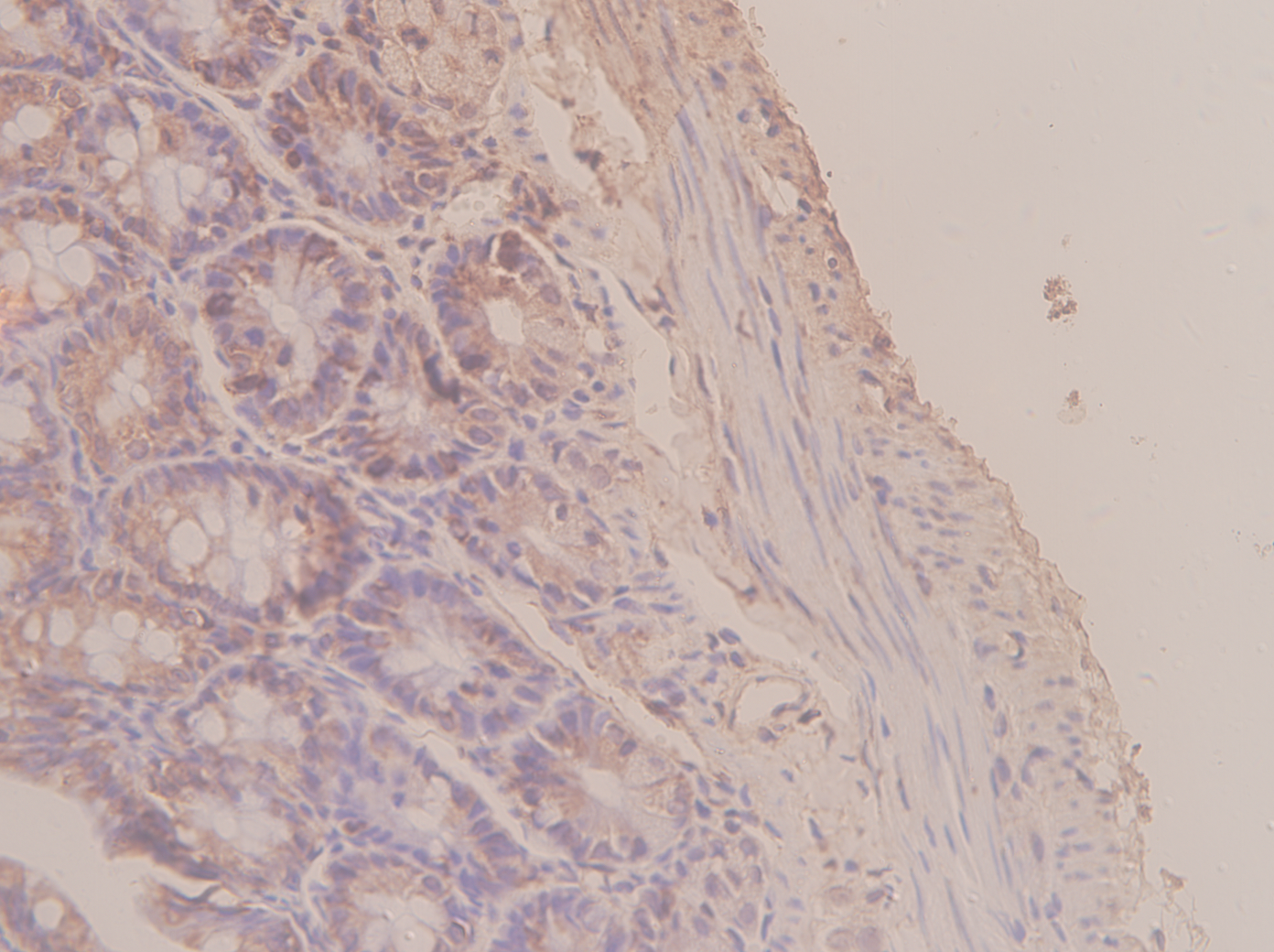

Supplement: DATA SHEET S4 — Original data for immunohistochemistry of intestinal COX-2. [file Data_Sheet_4.ZIP › FIG6A/COX-2/control/control-2.tif]

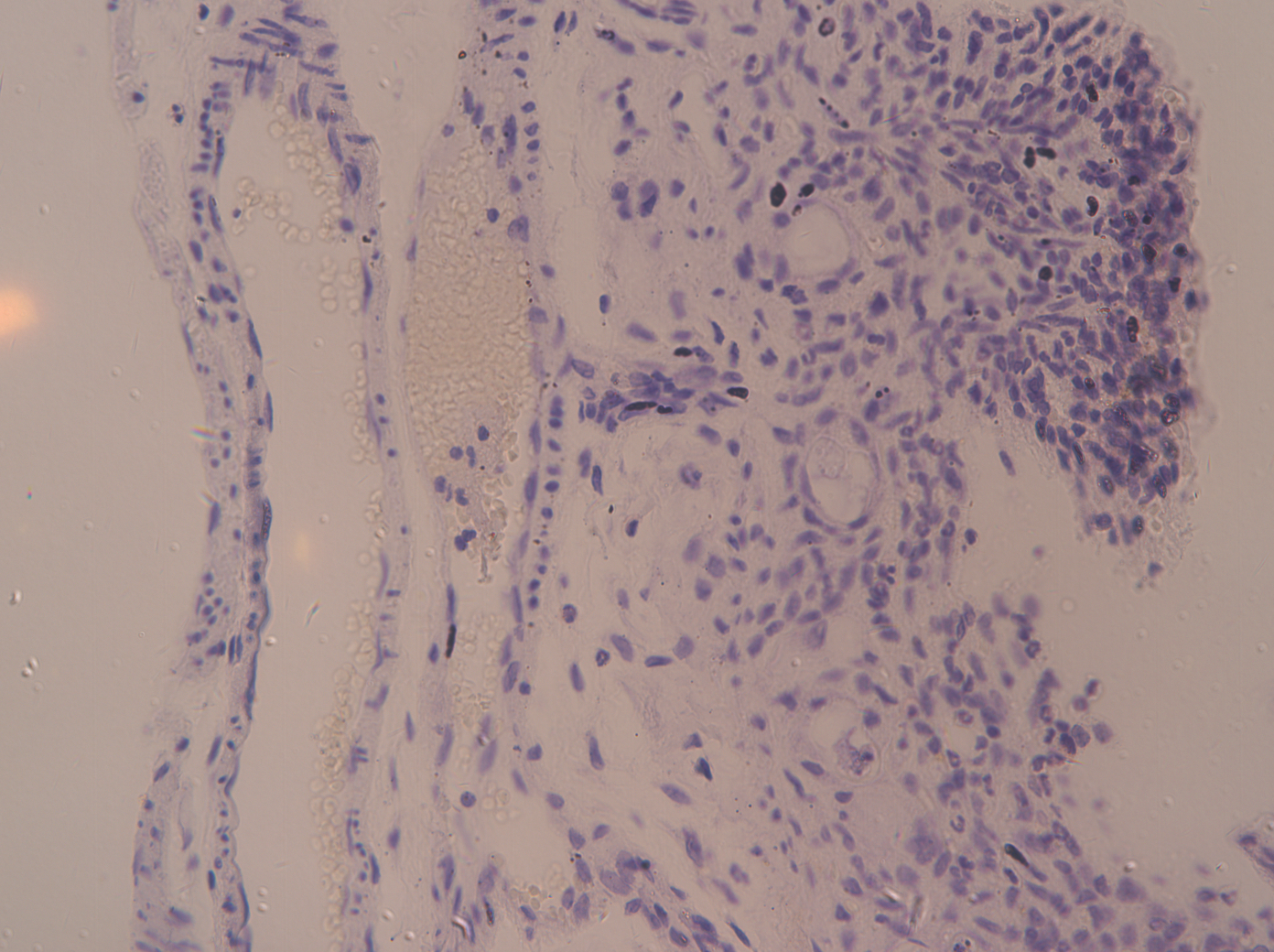

Supplement: DATA SHEET S4 — Original data for immunohistochemistry of intestinal COX-2. [file Data_Sheet_4.ZIP › FIG6A/COX-2/control/control-3.tif]
