# Supplementary material for: Protective Effects of Magnesium Glycyrrhizinate on Methotrexate-Induced Hepatotoxicity and Intestinal Toxicity May Be by Reducing COX-2
Source: Front Pharmacol. 2019 Mar 25;10:119. doi: 10.3389/fphar.2019.00119 (PMC6444054; doi:10.3389/fphar.2019.00119)
Supplement: DATA SHEETS S5, S6 — Standard biosecurity and institutional safety procedures. [file Data_Sheet_5.PDF]

动物实验福利伦理审查记录

申请编号	ACU-17 (20151124) A		批准编号	ACU-17 (20151125)	
试验名称	基于多种化疗药致肝损伤模型探讨甘美保肝作用机制		专题代号		
专题负责人	陆茵		专业/职称	教授	
	岗位证书编号				
拟进动物情况	品种品系：大鼠，Wistar		等级	√ SPF 清洁级 普通级	
	数量： 102 只（♂*102* 只；♀ 0 只）		规格	♂ *180-220g* ♀	
	申请日期： 2015 年 12 月 25 日		接收日期： 2015 年 12 月 30 日		
			结束日期： 2016 年 01 月 13 日		
课题执行人 1	曹玉珠	部门	药学院	专业/职称	药理学
岗位证书编号	2132213				
课题执行人 2		部门		专业/职称	
岗位证书编号					
课题执行人 3		部门		专业/职称	
岗位证书编号					
课题执行人 4		部门		专业/职称	
岗位证书编号					
课题执行人 5		部门		专业/职称	
岗位证书编号					
课题执行人 6		部门		专业/职称	
岗位证书编号					
课题委托单位				负责人	
联系电话		传真		邮 编	

实验内容概述，包括动物实验目的、意义、必要性、实验动物用途、饲养管理、实验处置、处死动物的方法等涉及动物伦理问题的详细论述。（可采用附件形式）

见附件
