# Supplementary material for: Protective Effects of Magnesium Glycyrrhizinate on Methotrexate-Induced Hepatotoxicity and Intestinal Toxicity May Be by Reducing COX-2
Source: Front Pharmacol. 2019 Mar 25;10:119. doi: 10.3389/fphar.2019.00119 (PMC6444054; doi:10.3389/fphar.2019.00119)
Supplement: Supplementary file 12 [file Data_Sheet_6.PDF]

申请人申明：我将自愿遵守实验动物福利伦理原则，随时接受实验动物伦理委员会的监督与检查，如违反规定，自愿接受处罚。

申请人签名：陆茵 2015 年 11 月 24日

依据动物保护和福利原则提出的问题及整改意见：

无

专题负责人确认签名： 年 月 日

伦理审查结果	实验动物伦理委员会 （ 5 人）同意 （ 0 人）不同意

实验动物伦理委员会意见

同意

主任（副主任）签名 王[illegible] 2015 年 11 月 25 日

备 注：
